# Bis(phenylethynyl)benzenes enable stable visible-to-ultraviolet sensitized triplet–triplet annihilation upconversion

**DOI:** 10.1039/d5tc02434j

**Published:** 2025-08-12

**Authors:** Davide Lardani, Alessandra Ronchi, Xueqian Hu, Angelo Monguzzi, Christoph Weder

**Affiliations:** a Adolphe Merkle Institute, University of Fribourg Chemin des Verdiers 4 1700 Fribourg Switzerland christoph.weder@unifr.ch; b Department of Material Science, University of Milano-Bicocca Via Roberto Cozzi 55 20125 Milan Italy angelo.monguzzi@unimib.it

## Abstract

Ultraviolet (UV) light plays a central role in applications ranging from photochemistry to sterilization and water treatment. However, its low abundance in sunlight (∼10%) limits the direct solar use of UV-driven processes. Sensitized triplet–triplet annihilation upconversion (TTA-UC) offers a promising route to generate UV light from visible light under low-power excitation. Yet, molecular systems capable of efficient visible-to-UV TTA-UC remain scarce. Here, we demonstrate that 1,4-bis(phenylethynyl)benzene (BPEB) and its alkoxylated derivative serve as efficient UV-emitting annihilators when paired with the visible-light sensitizer Ir(ppy)_3_ in toluene solution. These systems achieve upconverted emission centered at 380 nm, with anti-Stokes shifts exceeding 0.6 eV with respect to excitation energy and threshold excitation intensities as low as 11.5 mW cm^−2^. Spectroscopic studies suggest that modulation of high-energy excited-state dynamics plays a key role in optimizing upconversion performance. By broadening the molecular design space of UV-emitting annihilators beyond traditional polycyclic aromatics, this study provides a foundation for future development of low-intensity visible-to-UV TTA-UC systems. These findings expand the molecular toolkit for photonic applications where UV emission from ambient light is required.

## Abstract 2

Ultraviolet (UV) light is relevant for applications ranging from photochemistry to sterilization and water treatment. Triplet–triplet annihilation upconversion is an attractive process to convert low-power visible into UV light.

## Graphical abstract

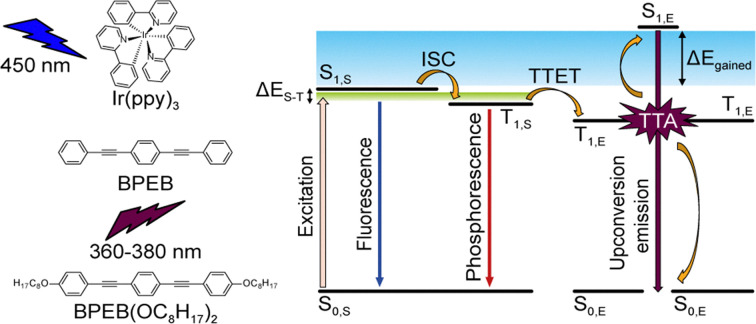

## 1. Introduction

**Fig. 1:**
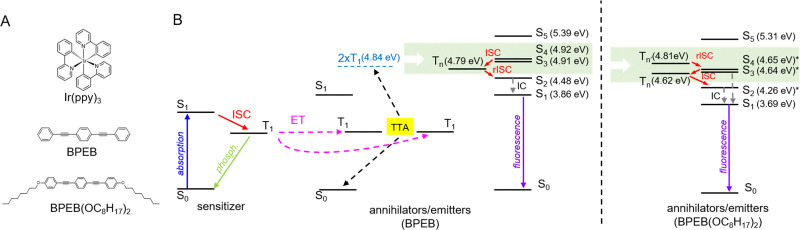
(A) Chemical structure of the iridium-based sensitizer Ir(ppy)_3_ and the UV emitters BPEB and BPEB(OC_8_H_17_)_2_. (B) Simplified Jablonski diagram illustrating the various photophysical steps at play in the TTA-UC process. The dashed arrows indicate Dexter energy transfer (ET), triplet–triplet annihilation (TTA), and internal conversion (IC). The red arrows mark the (reverse) intersystem crossing (ISC) steps both on the sensitizers and on the annihilators. The energies of the excited singlet states S_*n*_ shown for BPEB were taken from ref. 36. The T_*n*_ state energy was determined by combining the results of phosphorescence and transient absorption experiments (Fig. 2 and 5B). The shaded area highlights the electronic states potentially accessible to the triplet pair encounter complex during TTA. The energies of the excited singlet states S_*n*_ of BPEB(OC_8_H_17_)_2_ were calculated considering the redshift of 0.15 eV with respect to BPEB in the absorption and photoluminescence spectra in Fig. 2.

Ultraviolet (UV) photons power photochemical reactions in countless applications, including photocatalysis,^1^ water splitting for hydrogen production,^2,3^ bond activation,^4^ photopolymerization,^5,6^ and pollutant decomposition in water purification.^7–9^ Often, powerful UV light sources are employed whose intense light can also cause photodegradation.^10–13^ Moreover, UV lamps suffer from limited stability, their energy consumption is high, and their radiation poses safety problems.^14^ A possible strategy to address these issues is to exploit solar irradiation, but the UV portion of solar radiation is only 10%. This limitation can be overcome by methods that allow harnessing and converting visible (Vis) into UV light, including triplet–triplet annihilation (TTA-UC).^15,16^ TTA-UC is a nonlinear process based on the interplay between a triplet sensitizer and an annihilator/emitter. The sensitizer harvests the incident energy by absorption; the singlet excited states thus generated are converted into long-lived triplet states through intersystem crossing (ISC). The energy is then transferred *via* a short-range bimolecular Dexter energy transfer (ET) process to the triplet states of the emitter. Upon collision, two triplet excitons can annihilate and produce one singlet exciton, which decays radiatively and emits a high-energy photon (Fig. 1). If the electronic levels of the sensitizer/emitter pair are well matched, this mechanism proceeds with excellent yield under low excitation power density and with non-coherent photons, allowing operation under solar irradiance.^17^ Thus, TTA-UC represents, a priori, an attractive approach to producing UV photons by capturing the visible portion of the solar spectrum. While Vis-to-Vis TTA-UC has been extensively studied, research on UV-emitting upconverters is only emerging.^16,18^ Since the first studies by Castellano and co-workers,^19^ several Vis-to-UV TTA-UC systems have been developed using a variety of sensitizers, including all-organic as well as metal-based molecules,^16,20,21^ semiconductors, and perovskites,^22–26^ demonstrating the usefulness of TTA-UC also in this spectral region. Several proof-of-concept studies have shown that the upconverted UV emission can trigger specific reactions, driven by external visible light.^18,27–29^ Although performance levels comparable to those achieved with visible emitters have been reported, the palette of UV-light emitting triplet annihilators remains limited to a few candidates, which include 2,5-diphenyloxazole (PPO) and naphthalene derivatives, such as 1,4-bis((triisopropylsilyl)ethynyl)naphthalene (TIPS-Naph).^19,20,22,30^ One of the main causes of this limitation is the poor stability of the emitter. For example, in our hands, the widely used TIPS-Naph proved to degrade rapidly when combined with Ir(C6)_2_(acac) as triplet sensitizer (SI, Section S8). Another critical problem is the challenging design of conjugated UV-emitting molecules with an electronic energy level distribution that allows the efficient generation of singlet excitons upon TTA. Indeed, a prerequisite for efficient TTA is that the triplet energy level T_1_ is at least half of that of the first excited singlet S_1_, but lower than the S_2_ and T_2_ energies to avoid energy losses by internal conversion.^31,32^ A recently reported strategy to achieve the formation of singlet excited states is to make use of intersystem crossing pathways between resonant hot excited states (Fig. 1B).^33^ If the ISC between the hot triplet states T_*n*_ to the hot singlet states S_*n*_ is fast enough to surpass the T_*n*_ → T_1_ internal conversion, the T_*n*_ state energy can produce more emissive singlet states. This mechanism can be useful in OLED and scintillation devices, where the excitons produce simultaneously singlet and triplet states according to their spin multiplicity. This mechanism may also represent a possible pathway to improve the TTA-UC yield when the annihilators/emitters employed do not have the ideal energy level distribution of T_2_ ≫ 2T_1_ > S_1_, which promotes the relaxation of the triplet pair encounter complex 〈T_1_ + T_1_〉 to the emissive singlet state S_1_.^34^

Here, we show that the alkoxylation of 1,4-bis(phenylethynyl)benzene (BPEB) affects the interplay between singlet and triplet states. We investigate the upconversion properties of the alkoxylated derivative (BPEB(OC_8_H_17_)_2_)^35^ and the parent BPEB in combination with the iridium complex tris(2-phenylpyridine)iridium (Ir(ppy)_3_) as sensitizer. The results demonstrate that BPEB(OC_8_H_17_)_2_ shows a stable Vis-to-UV photon upconversion, with a two-fold higher ability to generate singlets upon TTA with respect to BPEB that we ascribe to a better interplay between hot states. These results underscore the potential of the BPEB platform as efficient UV emitters in TTA-UC and highlight the impact of seemingly minor molecular changes on the excited-state energy and interaction parameters.

## 2. Results and discussion

### 2.1. Photophysical characterization

**Fig. 2:**
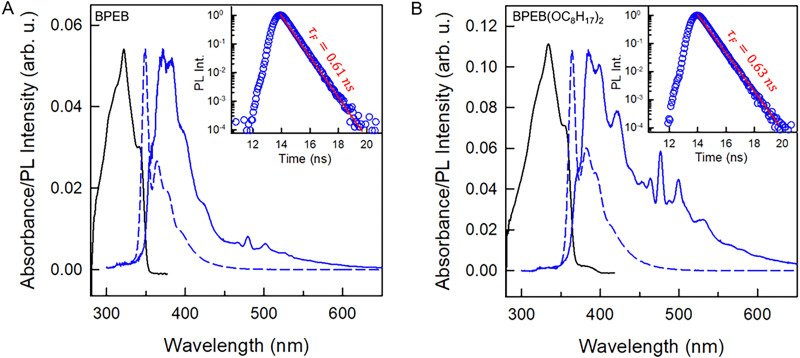
Absorption (solid black lines), room temperature photoluminescence (PL) (dashed blue lines), and PL spectra recorded at 77 K (solid blue lines) of (A) BPEB (*c* = 1.2 × 10^−5^ M) and (B) BPEB(OC_8_H_17_)_2_ (*c* = 1.7 × 10^−5^ M) in toluene. The PL spectra were acquired under excitation with a continuous wave (cw) laser operated at 266 nm. The insets show the room-temperature PL intensity decay curves detected at 360 nm recorded under excitation with a pulsed laser operated at 340 nm, along with single exponential decay fits to the data (red solid lines).

Ir(ppy)_3_ and BPEB are both commercial, while BPEB(OC_8_H_17_)_2_ was specifically synthesized as reported previously.^35^ We first investigated the photophysical properties of the two emitters in dilute toluene solutions (*c* = 1.2 × 10^−5^ M for BPEB, 1.7 × 10^−5^ M for BPEB(OC_8_H_17_)_2_) to reveal the effects associated with the alkoxylation of BPEB. Fig. 2 shows the absorption spectra of BPEB (panel A) and BPEB(OC_8_H_17_)_2_ (panel B), along with their photoluminescence spectra recorded under a continuous wave (cw) excitation at 266 nm at room temperature. A slight redshift of 15 nm (∼0.15 eV, Fig. S2, SI) in both the absorption and photoluminescence spectra is observed for BPEB(OC_8_H_17_)_2_. Although both molecules show the same fluorescence lifetime *τ*_F_ ∼ 0.62 ns (Fig. 2, inset),^37^ the fluorescence quantum yield *Φ*_F_, defined as the ratio of emitted and absorbed photons (SI, Section S2), is 1.00 ± 0.05 for BPEB and 0.87 ± 0.05 for BPEB(OC_8_H_17_)_2_. This result suggests the presence of fast competitive non-radiative deactivation channels in BPEB(OC_8_H_17_)_2_ that partially quench the excited singlet state.^38^ The solutions were frozen at 77 K to monitor the low-temperature phosphorescence from the dyes' triplet state (Fig. 2). The emission spectra change quite significantly due to suppression of vibrational coupling.^36^ For both dyes, a broad shoulder is seen in the green spectral region peaked at 511 nm (2.42 eV, Fig. S3, SI), which is attributed to triplet phosphorescence.^39^ This green emission has a characteristic lifetime of ∼1.6 ms, consistent with the slow radiative recombination of the dipole-forbidden T_1_–S_0_ transition (Fig. S4, SI).^40^ Notably, its intensity is much higher for BPEB(OC_8_H_17_)_2_ than for BPEB, which suggests a more efficient intersystem crossing for the modified emitter, in agreement with its lower *Φ*_F_. We further explored how the optical properties of the emitters change if the concentration is increased to *c* = 5 × 10^−3^ M in toluene, as high concentrations are generally required for TTA-UC to maximize the sensitization of triplet excitons and their annihilation rate. While no difference can be observed at the low-energy edge of the absorption spectra (Fig. S5, SI), which indicates the absence of significant aggregation, the high concentration emission spectra are clearly redshifted with respect to the diluted solutions, and the *Φ*_F_ drops to 0.57 for BPEB and 0.36 for BPEB(OC_8_H_17_)_2_. This reduction is attributed to a strong re-absorption of the photoluminescence (Fig. S6, SI).

**Fig. 3:**
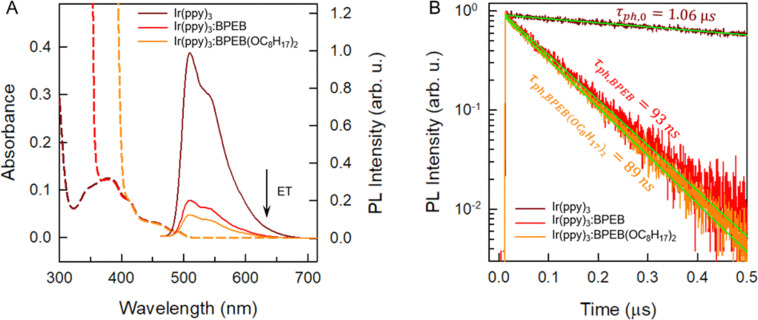
(A) Absorption (dashed lines) and PL (solid lines) spectra of toluene solutions of Ir(ppy)_3_ (brown), Ir(ppy)_3_ and BPEB (red), and Ir(ppy)_3_ and BPEB(OC_8_H_17_)_2_ (orange). The PL spectra were recorded under excitation with a continuous wave laser operated at 440 nm, *c*(Ir(ppy)_3_) = 2 × 10^−4^ M and *c*(BPEB or BPEB(OC_8_H_17_)_2_) = 5 × 10^−3^ M. (B) PL decay curves of the same solutions detected at 510 nm, recorded under excitation with a pulsed laser operated at 405 nm. Green solid lines are single exponential fits to the data.

We next combined the emitters with the phosphorescent complex (Ir(ppy)_3_) and investigated the TTA-UC process of these systems. By virtue of the heavy-metal effect, the intersystem crossing efficiency *Φ*_isc_ = 0.97 of Ir(ppy)_3_ is almost unity,^41^*i.e.*, singlet excitons created upon direct photon absorption almost quantitatively relax to the triplet state (Fig. 1B) and are at disposal for energy transfer to the emitters. The Ir(ppy)_3_ phosphorescence shows a maximum at 517 nm. This corresponds to a triplet energy of ∼2.40 eV, indicating a good resonance with the emitters’ triplets that should make the ET feasible (Fig. 1B). To assess the triplet sensitization from Ir(ppy)_3_, we studied how the presence of BPEB or BPEB(OC_8_H_17_)_2_ affects its phosphorescence. For this purpose, we analyzed a reference solution of Ir(ppy)_3_ in toluene (*c* = 2 × 10^−4^ M), as well as solutions containing Ir(ppy)_3_ (*c* = 2 × 10^−4^ M) and either BPEB or BPEB(OC_8_H_17_)_2_ (*c* = 5 × 10^−3^ M). Fig. 3A shows the absorption and photoluminescence spectra of these samples acquired under cw excitation of the sensitizer at 440 nm. As expected, the sensitizer phosphorescence is considerably quenched in the presence of emitters, indicative of triplet–triplet energy transfer. Moreover, as shown in Fig. 3B, the sensitizer triplet lifetime is reduced from *τ*^0^_Ph_ = 1.06 μs without emitter to *τ*_Ph_ = 93 ns for BPEB and to *τ*_Ph_ = 89 ns for BPEB(OC_8_H_17_)_2_. The energy transfer yield *Φ*_ET_ can be quantified by comparing the sensitizer phosphorescence intensities with (*I*_Ph_) and without (*I*^0^_Ph_) emitters or from the comparison of the phosphorescence lifetimes according to:1
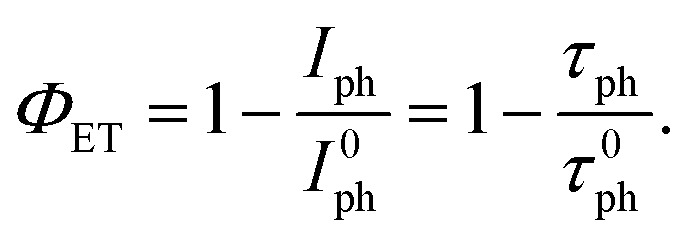


The analysis of the intensity data according to eqn (1) yields *Φ*_ET_ = 0.82 for the Ir(ppy)_3_:BPEB pair, and *Φ*_ET_ = 0.88 for the Ir(ppy)_3_:BPEB(OC_8_H_17_)_2_ pair, while values of 0.88 (Ir(ppy)_3_:BPEB) and 0.90 (Ir(ppy)_3_:BPEB(OC_8_H_17_)_2_) were determined from the time-resolved measurements. These findings demonstrate that Ir(ppy)_3_ is an efficient triplet sensitizer for both dyes. More in detail, the residual phosphorescence decay shows a long-time emission tail that is typical of back-energy transfer from the emitter to the sensitizer (Fig. S7, SI). Nevertheless, its contribution to the decay is <1% compared to forward ET, *i.e.*, its effect on the sensitizer's residual emission under steady-state conditions is negligible and does not appreciably affect the overall upconversion process.^42^ This fact and the observed high *Φ*_ET_ are indicative of an appropriate energy resonance between the sensitizer and emitter triplet energies that enable the transfer process. Moreover, the comparable*Φ*_ET_ observed for both emitters confirm that the derivatization does not significantly affect their triplet energy (Fig. 2 and Fig. S3, SI).

### 2.2. Upconversion properties

**Fig. 4:**
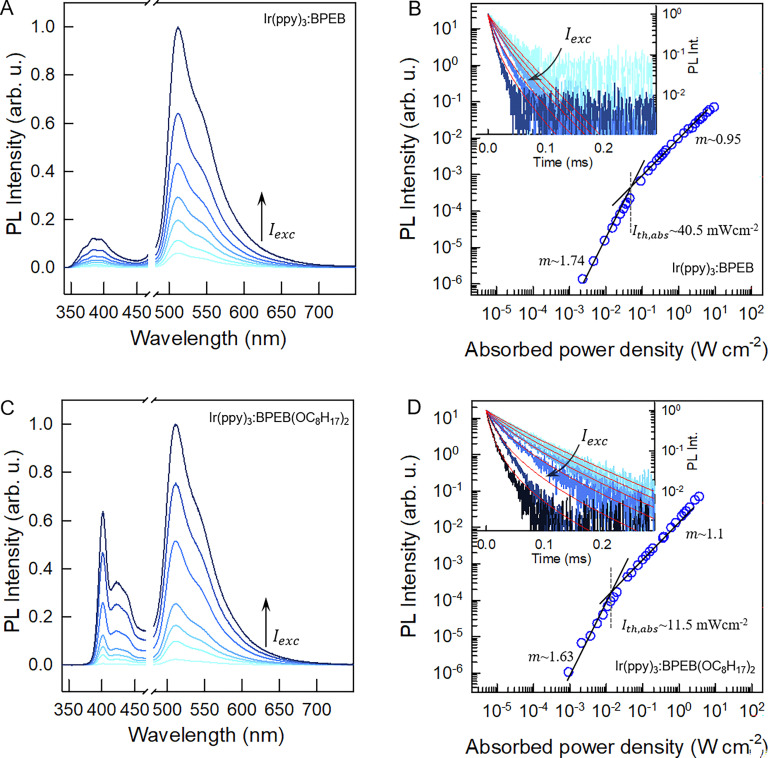
(A) and (C) Upconverted PL spectra of toluene solutions of (A) Ir(ppy)_3_:BPEB and (C) Ir(ppy)_3_:BPEB(OC_8_H_17_)_2_ acquired under cw excitation at 473 nm with increasing excitation power density *I*_exc_. For clarity, the scattered excitation light was removed (breaks). (B) and (D) Integrated upconverted PL intensity of the same (B) Ir(ppy)_3_:BPEB and (D) Ir(ppy)_3_:BPEB(OC_8_H_17_)_2_ solutions as a function of the absorbed power density at 473 nm *I*_exc,abs_. The solid black lines indicate the quadratic and linear regimes, and the vertical dashed lines mark the absorbed threshold intensities *I*_th,abs_. The insets show the decay curves of the upconverted emission intensity of the two systems under increasing excitation intensity, as indicated by the arrows. The red solid lines are fits of the data according to eqn (4). All experiments were carried out with *c*(Ir(ppy)_3_) = 2 × 10^−4^ M and *c*(BPEB or BPEB(OC_8_H_17_)_2_) = 5 × 10^−3^ M.

Once populated, the emitter triplets can recombine following different paths, mainly relaxing non-radiatively to their ground state or undergoing TTA. The latter mechanism leads to the formation of a triplet pair encounter complex 〈T_1_ + T_1_〉, which can evolve to an excited singlet, triplet, or quintet state, with a relative probability given by the spin multiplicity and by the molecular electronic structure.^43–45^ In the description of TTA processes, this probability is referred to as a statistical *f* factor. Because only the excited singlet can decay radiatively to the ground state, a high *f* value is pivotal for a high TTA-UC quantum yield *Φ*_UC_, which can be expressed as:^46^2
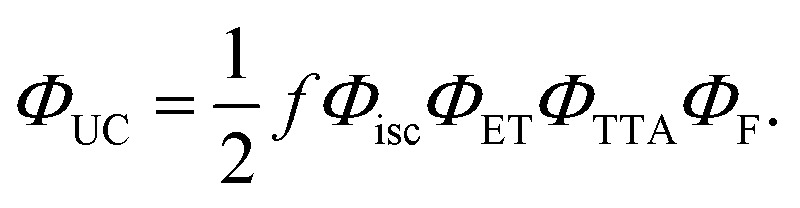
where *Φ*_isc_ is the sensitizer's ISC yield and *Φ*_F_ is the emitter fluorescence quantum yield at the concentration employed. Note that according to eqn (2), the maximum value of *Φ*_UC_ is 0.5. All parameters in eqn (2) are generally constant for a given sample, except for the TTA yield *Φ*_TTA_, which depends on the concentration of the triplets set by the excitation intensity.^47^ This translates into the peculiar quadratic-to-linear dependence of the TTA-UC emission intensity *I*_UC_ on the excitation intensity *I*_exc_. If the triplet density is too low to enable sufficient exciton collision, they mainly decay non-radiatively, and a quadratic relation *I*_UC_ ∝ *I*_exc_^2^ is observed. On the other hand, at high triplet density it is more probable for the triplets to undergo TTA rather than to spontaneously decay, and a linear correlation *I*_UC_ ∝ *I*_exc_ is observed. The excitation threshold intensity *I*_th_, at which the triplets have the same probability to decay spontaneously or by TTA, divides the two regimes. Thus, *I*_th_ corresponds to the excitation intensity at which the asymptotic quadratic and linear regimes cross in a logarithmic plot and half of the maximum *Φ*_UC_ is reached. It can be demonstrated that *I*_th_ can be approximated by:3
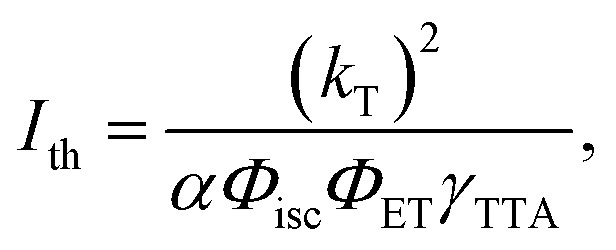
where *k*_T_ is the emitter triplet decay rate, *α* is the absorption coefficient at the excitation wavelength, and *γ*_TTA_ is the second-order rate constant of the TTA process.^47^ Since it is generally desirable to minimize *I*_th_, eqn (3) is an important tool as it highlights the parameters that govern *I*_th_ and points out which molecular characteristics and system parameters can be used to lower it as much as possible. To assess the TTA-UC performance of the Ir(ppy)_3_:BPEB and Ir(ppy)_3_:BPEB(OC_8_H_17_)_2_ pairs, we analyzed the upconversion figures of merit, *Φ*_UC_ and *I*_th_, using the same toluene solutions as employed for the energy transfer experiments discussed above. Fig. 4A and C show the photoluminescence spectra of the Ir(ppy)_3_:BPEB and of the Ir(ppy)_3_:BPEB(OC_8_H_17_)_2_ pair measured as a function of the absorbed excitation intensity under cw laser excitation at 473 nm, *i.e.*, at a wavelength where only the sensitizer absorbs (the absorbance at this wavelength is 0.0186 and 0.0192 for Ir(ppy)_3_:BPEB and Ir(ppy)_3_:BPEB(OC_8_H_17_)_2_, respectively). The UC emission intensity of a solution of Ir(ppy)_3_:BPEB shows a slight reduction of 15% after 1 hour of high-intensity excitation, whereas the upconverted emission of Ir(ppy)_3_:BPEB(OC_8_H_17_)_2_ remains unchanged (Fig. S22, SI). Thus, the bis(phenylethynyl)benzene-based annihilators surpass the photostability of other annihilators/emitters for Vis-to-UV TTA-UC, including TIPS-Naph (Fig. S21, SI). Fig. 4B and D show plots of the integrated UC emission intensity *I*_UC_ (*λ* < 450 nm) of solutions containing BPEB or BPEB(OC_8_H_17_)_2_ as a function of the absorbed excitation intensity *I*_exc,abs_. Both *I*_UC_*vs. I*_exc,abs_ plots show the expected quadratic and linear regimes, and their intersections mark the absorbed power threshold intensity *I*_th,abs_. Intriguingly, this value differs considerably between the two systems, which are characterized by an estimated average *I*_th,abs_ ∼ 40.5 mW cm^−2^ (BPEB) and *I*_th,abs_ ∼ 11.5 mW cm^−2^ (BPEB(OC_8_H_17_)_2_) (Fig. S8, SI). Both sensitizer/emitter pairs exhibit similar values of *α*, *Φ*_isc_ and *Φ*_ET_, and it thus follows from eqn (3) that the four-fold difference in *I*_th_ must be related to the emitter's triplet lifetime at room temperature. To validate this conclusion, we recorded the UC emission decays under modulated laser excitation as a function of the excitation intensity *I*_exc_ as shown in the insets of Fig. 4B and D (Methods in SI). The decay traces of the UC signal behave as expected for the TTA-UC dynamics, *i.e.*, the upconverted emission intensity *I*_UC_(*t*) decays according to:^31,43^4
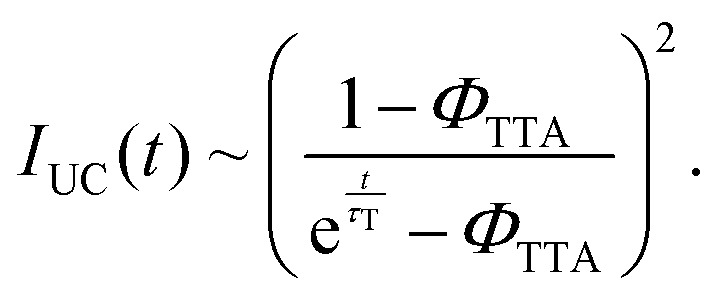
Here *Φ*_TTA_ is the TTA efficiency at *t* = 0, *i.e.*, immediately after switching off the modulated laser, which is governed by the initial triplet population and thus the excitation intensity. The UC decay curves change from a single exponential decay at low excitation intensity, *i.e.*, when TTA is unlikely to occur (*Φ*_TTA_ ≪ 1), to a faster dynamic at high excitation intensity, as the triplet density increases and the TTA process becomes more and more effective. By fitting the decay curve at the lowest excitation intensity with a single exponential function, we estimate an emitter triplet lifetime *τ*_T_ of 62 μs for BPEB and 150 μs for BPEB(OC_8_H_17_)_2_. This is a crucial result, as it suggests that the structural modification of BPEB with peripheral alkoxy groups protects the lowest triplet state from non-radiative decay pathways by reducing the number and intensities of vibrational modes allowed at room temperature (Fig. S4, SI), without affecting the electronic structure. Thus, BPEB(OC_8_H_17_)_2_ displays a higher *τ*_T_ and therefore a lower excitation threshold (eqn (3)). We estimated the maximum UC efficiency *Φ*_UC_ by using the residual sensitizer emission in the linear regime (*I*_exc,abs_ ∼ 0.4 Wcm^−2^ and *I*_exc,abs_ ∼ 0.07 Wcm^−2^ respectively) as reference (eqn (S2), SI), which yields *Φ*_UC_ ∼ 0.024 for BPEB and *Φ*_UC_ ∼ 0.023 for BPEB(OC_8_H_17_)_2_. The power-dependent *Φ*_UC_ values were used to assess if and how the emitter's molecular structure influences the *f* factor (Fig. S8, SI). We first determined *Φ*_TTA_ at the same *I*_exc,abs_ used to estimate *Φ*_UC_ by fitting the UC decay curves in Fig. 4B and D with eqn (4) (Table S1, SI). Considering the high-concentration *Φ*_F_, eqn (2) affords average values of *f* ∼ 0.13 ± 0.03 for BPEB and *f* ∼ 0.24 ± 0.02 for BPEB(OC_8_H_17_)_2_. These values reflect that in BPEB(OC_8_H_17_)_2_, the population of the emitter's fluorescent excited singlet state after annihilation is considerably improved, doubling the singlet generation yield *via* TTA.

**Fig. 5:**
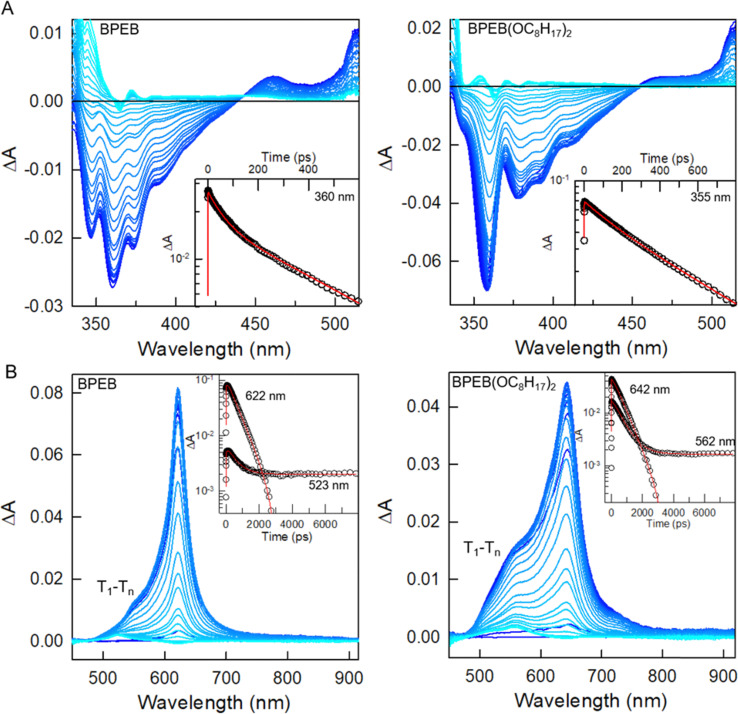
Ultrafast transient absorption (TA) spectra recorded under a 340 nm pump, monitoring the absorbance change Δ*A* employing a UV probe (panel (A)) or a Vis probe (panel (B)), relative to BPEB (left) and to BPEB(OC_8_H_17_)_2_ (right). The delay time increases from dark blue to light blue. The maximum time delay is *ca.* 8 ns. The insets show the TA kinetics for the wavelengths indicated by the labels. The kinetics in panel B were turned positive for clarity. The red lines are the fitting curves performed as reported in the SI, Section S4.

A possible explanation for this behavior is an enhanced interplay between the singlet and triplet hot states in BPEB(OC_8_H_17_)_2_. By considering that the energy of the triplet pair encounter complex 〈T_1_ + T_1_〉 is 2 × T_1_ = 4.84 eV, good resonances with S_3_ and S_4_ states of BPEB are apparent (Fig. 1B).^36^ Moreover, the transient absorption (TA) experiments discussed below (Fig. 5B) point out the presence of a T_*n*_ state at around 4.80 eV for both dyes and also at around 4.62 eV only for BPEB(OC_8_H_17_)_2_. These T_*n*_ states are highly resonant with 2 × T_1_ and therefore energetically fully accessible through TTA (Fig. 1B). This suggests that the singlet:triplet formation upon TTA does not present a preferential branching ratio, at least at room temperature. However, the large resonance between hot S_*n*_ and T_*n*_ states, which enables the exciton delocalization over the singlet and triplet manifolds, can induce a hot-state ISC, which is faster than internal and radiative recombination.^34,45^ This loop can be unbalanced towards the S_1_ state, from which fluorescence occurs, resulting in an overall higher apparent *f* factor. The unbalance towards the singlets manifold could be mediated by the S_2_ state, which lies slightly below T_*n*_ for both dyes (Fig. 1B). Considering the excited state energetic panorama and the observed enhanced ISC between low energy singlet and triplet states (Fig. 2), we speculate that an enhanced reversed ISC between T_*n*_ and S_*n*_ states in BPEB(OC_8_H_17_)_2_ is the crucial step that leads to a higher singlet generation compared to BPEB.

### 2.3. Transient absorption measurements

To support this hypothesis, we further investigate the interplay between singlet and triplet manifolds in BPEB(OC_8_H_17_)_2_ by ultrafast TA experiments. Fig. 5 shows the TA spectra of diluted BPEB and BPEB(OC_8_H_17_)_2_ solutions at incrementing delay times, acquired under a 340 nm pump and using a UV (Fig. 5A) or visible (Fig. 5B) light probe beam, respectively. The negative feature in the UV regime is consistent with a mixture of ground-state bleach (GSB) and stimulated emission (SE) (Fig. 2). As reflected by fits of the data (SI, Section S4 and Fig. S9), the TA in this spectral range decays with a fast initial component of about 80 ps for BPEB and 200 ps for BPEB(OC_8_H_17_)_2_, and a main slower component on the order of 600 ps, which is consistent with the photoluminescence lifetime reported in Fig. 2 (Tables S2 and S3, SI). Thus, the TA spectra clearly reflect the absorbance of the fluorescent photo-excited singlet state S_1_ (Fig. 1B). The TA in the Vis regime (Fig. 5B) also reflects two kinetically different processes. The feature around 630 nm decays with a characteristic time of *ca.* 600 ps, thus in agreement with fluorescence, and a rise time of 80 ps for BPEB and 200 ps for BPEB(OC_8_H_17_)_2_ (Fig. S10 and Tables S4, S5, SI). We ascribe this feature to absorption from S_1_ (possibly the S_1_–S_5_ transition in Fig. 1). Conversely, the feature around 500–550 nm does not completely decay within 8 ns. Moreover, it shows a rise time constant that is similar to the fast decay component observed in the GSB/SE kinetics. Therefore, we assign these features to a T_1_–T_*n*_ absorption, when T_1_ states are populated by ISC from the photo-excited S_1_. The increased initial relative intensity of the triplet absorption feature with respect to that of the singlet in BPEB(OC_8_H_17_)_2_ suggests an increased ISC efficiency. A possible origin of this enhanced ISC could be glimpsed looking at the spectral migration data reported in Fig. S11 (SI). Unlike BPEB, the GSB/SE feature of BPEB(OC_8_H_17_)_2_ shows a clear redshift of about 39 meV in the initial 100 ps. This suggests that after excitation, the BPEB(OC_8_H_17_)_2_ molecule undergoes a larger but slower reorganization to the relaxed S_1_ excited state. This is most probably due to the presence of the lateral chains, even if it is unlikely that they hinder the motion of the phenylethynyl groups.^48^ Therefore, we speculate that during this slow reorganization other pathways for ISC between the triplets and singlets manifolds may become available, resulting in a final larger T_1_ state population and also favoring the spin–flip between the largely resonant hot states of the system.

## 3. Conclusions

In summary, we have demonstrated the capability of BPEB and its alkyloxylated derivative BPEB(OC_8_H_17_)_2_ to serve as stable UV annihilators/emitters for visible-to-UV photon upconversion when combined with a suitable sensitizer. At room temperature, BPEB(OC_8_H_17_)_2_ shows long-living triplets and a higher singlet generation probability *f* through TTA with respect to the unmodified BPEB. In combination with the visible-light sensitizer Ir(ppy)_3_, these features permit blue to UV photon upconversion from 473 nm to 380 nm, with a net photon energy gain of 0.65 eV and a reduced excitation intensity threshold of 11.5 mW cm^−2^, comparable to the solar irradiance under AM1.5 conditions.

Interestingly, the modified annihilator shows an almost doubled efficiency of the singlet states generation through TTA compared to BPEB. The photoluminescence and excited state spectroscopy investigation performed indicates that the alkoxylation of BPEB enhances the interplay between singlet and triplet manifolds in BPEB(OC_8_H_17_)_2_. While a quantitative analysis is difficult, these findings suggest an enhanced ISC between its highly resonant T_*n*_ and S_*n*_ hot states. This effect favors the production of upconverted emissive singlet excitons and is at least partially responsible for the higher *f* value observed.

The obtained results confirm the versatility of BPEB and its derivatives as potential UV emitters with tailored properties. In the case of BPEB(OC_8_H_17_)_2_, the higher *f* value is counterbalanced by a lower *Φ*_F_, which limits the external TTA-UC yield, but the data show clearly that influencing the interactions of the high-energy excited states by simple structural changes is an attractive approach to develop new annihilators/emitters for TTA-UC.

## Author contributions

The data underlying this manuscript was written through contributions of all authors. All authors have given approval to the final version of the manuscript.

## Conflicts of interest

The authors declare no conflict of interest.

## Supplementary Material

TC-013-D5TC02434J-s001

## Data Availability

The dataset underlying the findings of this article can be found at https://doi.org/10.5281/zenodo.15705877. Supplementary information available: Experimental details and methods, photophysical studies, synthesis, NMR spectra. See DOI: https://doi.org/10.1039/d5tc02434j
