# Supplementary material for: Bis(phenylethynyl)benzenes enable stable visible-to-ultraviolet sensitized triplet–triplet annihilation upconversion
Source: J Mater Chem C Mater. 2025 Aug 12;13(36):18796–804. doi: 10.1039/d5tc02434j (PMC12362340; doi:10.1039/d5tc02434j)
Supplement: TC-013-D5TC02434J-s001 [file TC-013-D5TC02434J-s001.pdf]

Supplementary Information (SI) for Journal of Materials Chemistry C.
This journal is © The Royal Society of Chemistry 2025

Supporting Information

Bis(phenylethynyl)benzenes enable stable visible-to-ultraviolet sensitized triplet-triplet annihilation upconversion

*Davide Lardani[1+], Alessandra Ronchi[2+], Xueqian Hu[1], Angelo Monguzzi[*2], Christoph Weder[*1]*

[1] Adolphe Merkle Institute, University of Fribourg, Fribourg 1700, Switzerland

[2] Department of Material Science, University of Milano-Bicocca, Milan 20125, Italy

[+]These authors contributed equally.

Index:

1. Experimental details and methods page S2
2. Methods page S3
3. Additional data page S5
4. Ultrafast transient absorption kinetic analysis page S10
5. Transient absorption spectra analysis page S13
6. Synthesis page S13
7. NMR spectra page S15
8. Reference Vis-to-UV photon upconversion system page S18
9. Stability Test page S20
10. Supporting References page S21

1. Experimental details and methods

Materials: Unless stated otherwise, all the chemicals used for the experiments were purchased commercially and used without further purification: 1,4-Bis(phenylethynyl)benzene (BPEB), 97 %, Fluorochem; toluene, 99.8 %, Fisher Chemical; toluene, 99.8 %, Sigma Aldrich; tetrahydrofuran, 99.9 %, Sigma Aldrich; 1,4-bis((4-(octyloxy)phenyl)ethynyl)benzene (BPEB(OC_8H_{17})$_2$) was synthesized according to the procedure described in the Section *Synthesis* below.

Sample preparation: All samples were prepared in toluene, and the measurements were carried out in 0.1 cm optical path quartz cuvettes. For energy transfer, upconversion, and transient absorption measurements, all samples were prepared in a glove box, in a nitrogen atmosphere (oxygen concentration < 1 ppm), and carefully sealed to prevent oxygen contamination that could quench the triplet excitons.[1,2]

Chromatography: Thin-layer chromatography (TLC) was performed using ALUGRAM® Xtra SIL G/UV$_{254}$ pre-coated aluminum sheets (REF 818331) and a UVP Cambridge (UK) UVGL-58 Handheld UV Lamp, 6 W, 254/365 nm, was used for the detection of the compounds. The automatic liquid column chromatography Biotage Isolera™ One equipped with a BGB Scorpius Flash Cartridge (BSS2CF-W080), silica 60 Å, was used for the purification of 1-iodo-4-(octyloxy)benzene and BPEB(OC_8H_{17})$_2$.

NMR spectroscopy: A Bruker Avance DPX 400 spectrometer was used to record the ^1H (400.19 MHz) and ^{13}C (100.63 MHz) at 297.2 K in $CDCl_3$. ^1H-NMR and ^{13}C-NMR spectra were calibrated to the residual solvent peak of $CDCl_3$ (7.26 ppm and 77.36 ppm). The spectra were analyzed with the software MestReNova 11.0.1-17801. The chemical shifts (δ) are expressed in parts per million (ppm) with the coupling constant (J) in Hz.

Fourier Transform Infrared (FT-IR) Spectroscopy: Infrared spectra were recorded using a PerkinElmer Spectrum 65 FT-IR spectrometer equipped with an attenuated total reflection (ATR) accessory. Each spectrum was obtained by averaging four scans over the spectral range of 600 to 4000 cm^{-1}.

2. Methods

Optical absorption spectra were measured with an Agilent Cary 60 spectrometer in normal incidence conditions using 1 mm thick quartz cuvettes. Room temperature, steady-state photoluminescence (PL) spectra were carried out with a Varian Cary Eclipse fluorescence spectrometer, selecting the excitation wavelength from the emission of the integrated Xe lamp, and collecting the emitted light with a phototube. The spectra were corrected for the spectral response of the system.

The steady-state, power-dependent upconversion (UC) PL spectra were recorded using a DPSSL continuous-wave (cw) 473 nm laser (MBL- III-473-10mW), provided with TTL modulation, as excitation source. The emission was recorded with a charge-coupled device detector (Jobin-Yvon Syncerity) coupled to a monochromator (JobinYvon Triax 190) with a 300 lines/mm grating. The spectra reported are not corrected for the instrumental spectral response for clarity because of the noise introduced at the spectral blue-edge, but for the UC efficiency calculations the corrected spectra were used. To perform the power-dependent measurements, we varied the laser intensity using reflective power density neutral filters and measured it with an optical power meter (Thorlabs PM100USB, power sensor S120VC). The laser beam diameters were measured by the knife blade method.

The time-resolved power-dependent UC measurements were performed with the cw 473 nm laser, and the laser output was modulated with a square wave signal with a repetition rate of 500 Hz, using a TTi TG5011 wavefunction generator. The signal was recorded by a nitrogen cooled photomultiplier (Hamamatsu R5509-73) coupled with a high-speed amplifier (Hamamatsu C5594), a 74100 Cornerstone 260 1/4 m VIS-NIR monochromator (ORIEL), and a PCI plug-in multichannel scaler ORTEC 9353 100 ps time digitizer/MCS in time-correlated single photon counting (TCSPC) acquisition mode (time resolution ~400 ps).

The PL time-resolved measurements as a function of temperature were performed by using the 266 nm harmonic of Nd:YAG Continuum Minilite laser (10 ns pulse width) or a Laser-Export 355 nm pulsed laser (DTL-375QT) as excitation source according to the sample, collecting the emitted light with the same apparatus of time-resolved power-dependent UC measurements.

The UV emitters PL characteristic lifetimes were measured with a 340 nm ps-pulsed LED laser (EP-LED 340 Edinburgh Instruments), and the emitted light was collected with a FLS980 spectrometer (Edinburgh Instruments) coupled to a PicoHarp 300 hybrid photomultiplier tube working in TCSPC mode. The same experimental setup was also used for the time-resolved measurements for energy transfer studies but a 405 nm ps-pulsed diode laser (Edinburgh EPL405) was used. The overall time resolution of the setup was 300 ps.

The ultrafast transient absorption measurements were performed on Ultrafast Systems' Helios TA spectrometer. The laser source was a 10 W Ytterbium amplified laser operated at 1.875 kHz producing ~260 fs pulses at 1030 nm and coupled with an independently tunable optical parametric amplifier from the same supplier that produced the excitation pulses at 3.1 eV (400 nm). After passing the pump beam through a synchronous chopper phase-locked to the pulse train (0.938 kHz, blocking every other pump pulse), the pump fluence on the sample was modulated using a variable ND filter. With this setup the maxim time delay between pump and probe pulses is ca. 8 ns. The probe beam was a white light supercontinuum in the VIS or UV range.

Photoluminescence quantum yield: The photoluminescence quantum yields Φ_F of the molecules used were determined by comparing their photoluminescence spectra to that of a reference solution with known quantum yield Φ_{ref}, according to equation S1

$$\Phi_F = \Phi_{ref}\frac{I_s\ A_{ref}\ n_s^2}{I_{ref}\ A_s\ n_{ref}^2} \qquad \text{(S1)}$$

Here, *I* is the photoluminescence intensity of the sample under study (*s*) or of the reference (*ref*), *A* is the fraction of photons absorbed at the excitation wavelength, and *n* is the refractive index of the solvents used. The comparative measurements were performed under the same experimental conditions to be significant.

For the emitters' quantum yield, we employed a 2,5-diphenyloxazole (PPO, $\Phi_{ref} = 1$)[3] solution (c=3.7×10^{-5} M) in cyclohexane as a reference sample. To establish the Φ_{PL} of the sensitizers at the concentrations used for the UC studies in toluene, we employed a 9,10-diphenylanthracene (DPA, $\Phi_{ref} = 0.97$)[4] solution (c=10^{-4} M) in cyclohexane as reference. The measured phosphorescence quantum yield of Ir(ppy)$_3$ is 0.95, which is consistent with previous results.[5]

Upconversion quantum yield: Since it was not easy to find a proper reference to directly measure the upconversion efficiency Φ_{UC}, we estimated this value by comparing the upconverted emission and residual sensitizer emission intensities (I_{UC} and I_{sens}, respectively), in the I_{UC} vs excitation intensity linearity region. From the phosphorescence quantum yield of Ir(ppy)$_3$ in toluene Φ_{sens} at the concentration used in the upconverting solutions ($c = 2 \times 10^{-4}$ M), and knowing the energy transfer efficiency Φ_{ET}, we estimated the Φ_{UC} values using Eq. S2, which is a slightly modified version of equation S1,

$$\Phi_{UC} = \Phi_{sens} I_{UC} \frac{1 - \Phi_{ET}}{I_{sens}} . \quad \text{(S2)}$$

3. Additional data

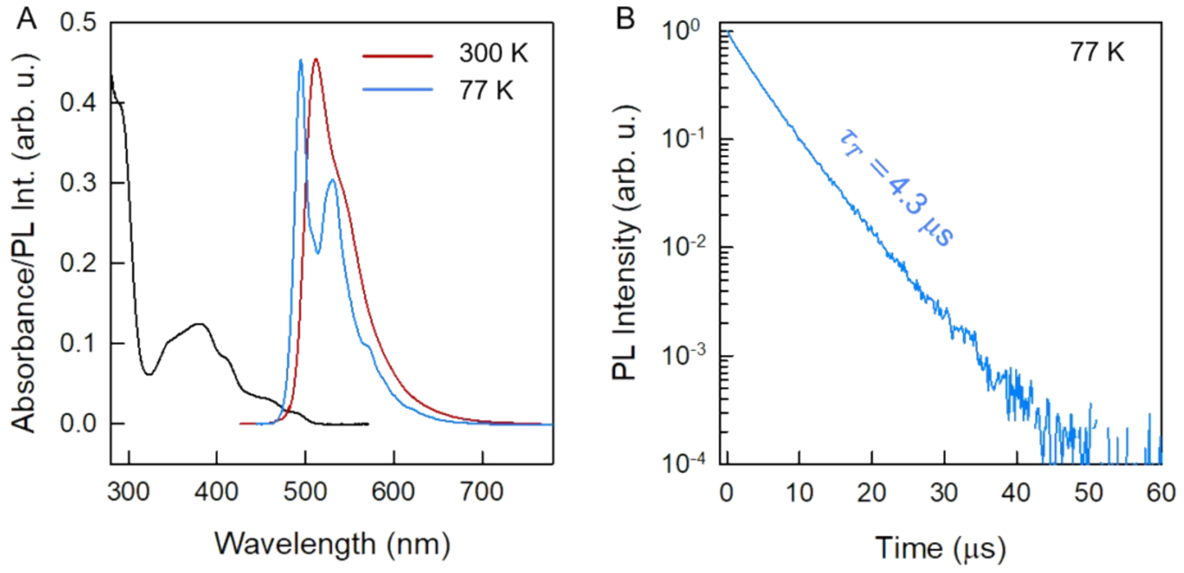

Figure S1. (A) Absorption (black line) and emission (red and blue lines) spectra of the $Ir(ppy)_3$ toluene solution (c=5×10^{-4} M). The red and blue traces show the PL spectra of the solution recorded at room-temperature and 77 K, respectively, under a 355 nm laser excitation(77 K). (B) Decay curve of the 505 nm triplet state emission at 77 K, recorded under a 355 nm pulsed laser excitation. The characteristic triplet lifetime $\tau_T =$ 4.3 μs was estimated as the effective time when the initial intensity drops to $^1/_e$.

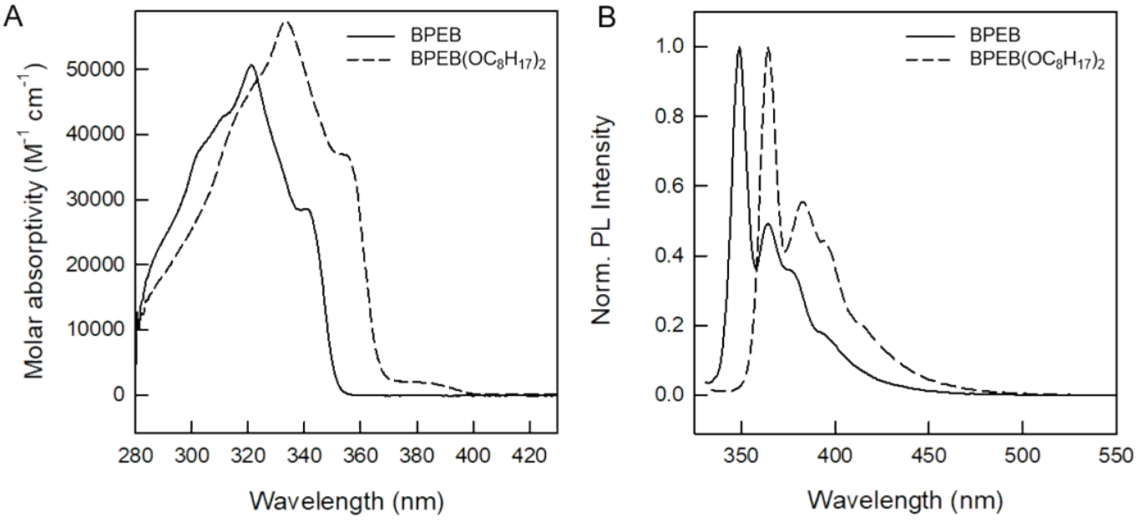

Figure S2. (A) Molar absorptivity of a diluted BPEB (solid line) and BPEB(OC_8H_{17})$_2$ (dashed line) solution (c=5×10^{-5} M) in toluene. The data were derived from the corresponding absorption spectra acquired in 1 mm cuvettes. (B) Normalized PL spectra of a diluted BPEB (solid line) and BPEB(OC_8H_{17})$_2$ (dashed line) solution (c=5×10^{-5} M) in toluene. Both absorption and PL spectra show a redshift of about 15 nm (~ 0.15 eV) between BPEB and BPEB(OC_8H_{17})$_2$.

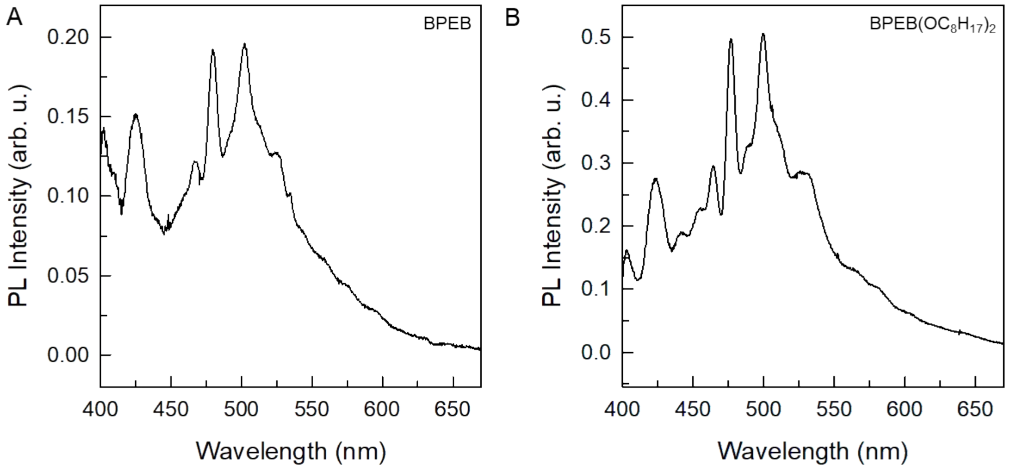

Figure S3. (A, B) Time gated phosphorescence spectra recorded after 15 ns for the dye solution in toluene (c(BPEB)=5×10^{-3} M) in panel A, and c(BPEB(OC_8H_{17})$_2$)=5×10^{-3} M in panel B at 77 K, under 266 nm laser excitation.

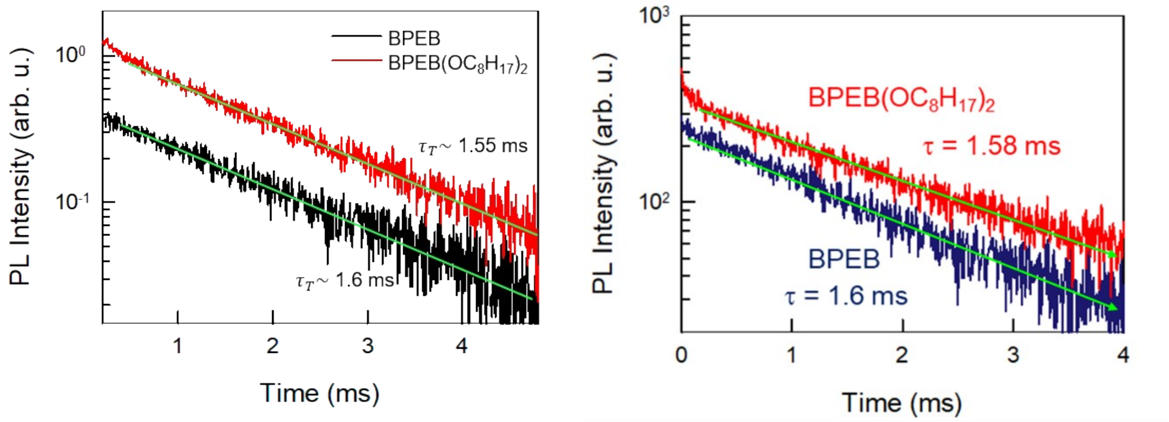

Figure S4. Phosphorescence decay curves recorded at 77 K at 511 nm (left) and 475 nm (right) under a pulsed laser operated at 266 nm, of a BPEB (black curve) and of a BPEB(OC_8H_{17})$_2$ (red curve) toluene solution. The low temperature triplet lifetimes reported were inferred from the single exponential fits (green lines).

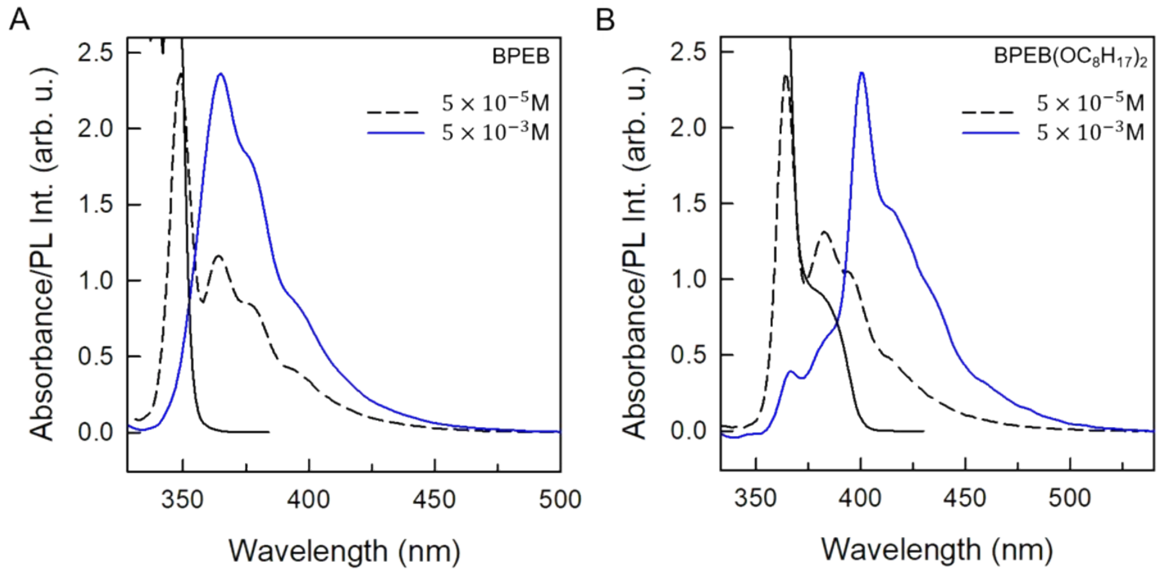

Figure S5. (A, B) Absorption spectrum (solid black curves) of a high concentration solution in toluene (*c*(BPEB)=5×10^{-3} M in A, *c*(BPEB(OC_8H_{17})$_2$)=5×10^{-3} M in B), along with the PL spectrum (blue) acquired under a cw 320 nm excitation. The dashed curves are the PL spectra of the corresponding diluted solutions (*c*(BPEB)=5×10^{-5} M) in A, and *c*(BPEB(OC_8H_{17})$_2$=5×10^{-5} M) in B.

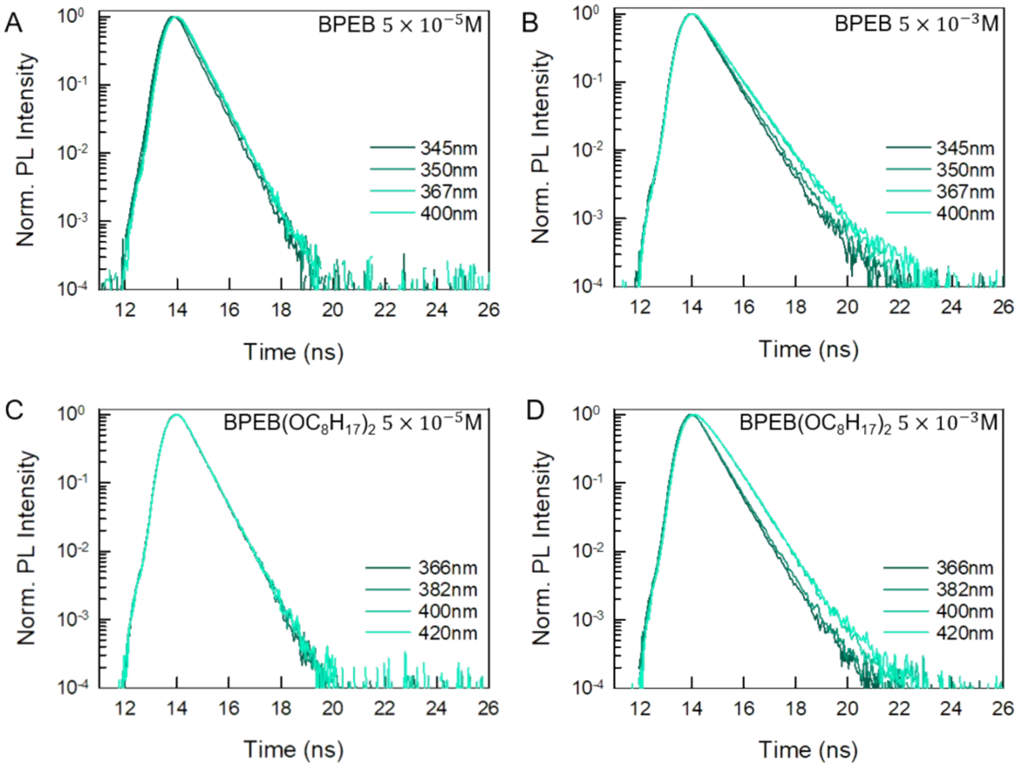

Figure S6. PL decay curves detected at selected wavelengths recorded under excitation with a pulsed laser operated at 340 nm, of a diluted (A, $c=5\times10^{-5}$ M) and concentrated (B, $c=5\times10^{-3}$ M) BPEB toluene solution, and of a diluted (C, $c=5\times10^{-5}$ M) and concentrated (D, $c=5\times10^{-3}$ M) BPEB(OC_8H_{17})$_2$ toluene solution. For both emitters the PL decay curves are independent of the emission wavelength in the diluted case, whereas in the concentrated samples the PL dynamics are slowed down and this behavior is accentuated towards longer wavelengths. The BPEB lifetime goes from 0.63 ns in the diluted solution to a maximum of 0.79 ns at 400 nm in the concentrated one. The BPEB(OC_8H_{17})$_2$ lifetime goes from 0.6 ns in the diluted solution to a maximum of 0.76 ns at 420 nm in the concentrated one. This behavior is consistent with reabsorption of the emitted light.

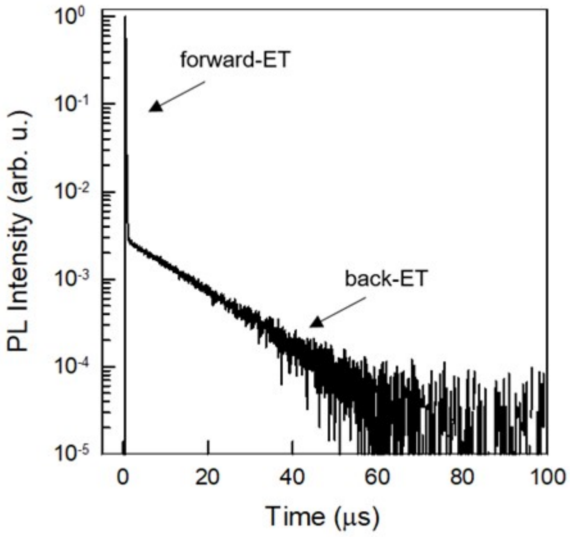

Figure S7. PL decay curve of the upconverting c(Ir(ppy)$_3$)$=2\times10^{-4}$ M and c(BPEB)$=5\times10^{-3}$ M solution detected at 510 nm, recorded under excitation with a pulsed laser operated at 405 nm. The decay curve shows a bi-exponential behaviour. The fast component is associated to forward ET, consistent with the data reported in Figure 3 in the main text, while the long slow component is associated to back ET from the emitter triplets to the sensitizer triplets.

Table S1. TTA yield values as a function of the absorbed excitation intensity derived from fitting the data with Eq. 4 (Φ_{TTA}) and from the lifetime of the delayed upconverted fluorescence measured as $\bar{\tau}_{uc} = 0.5\ \bar{\tau}_T = I_0/e$. $\bar{\Phi}_{TTA}$ is calculated as $1 - \bar{\tau}_T/\tau_T$.

	Exc. intensity (W cm^{-2})	Φ_{TTA}	τ_T (μs)	$\bar{\tau}_{uc}$ (μs)	$\bar{\tau}_T$ (μs)	$\bar{\Phi}_{TTA}$
BPEP	0.01	0.26	62	27	54	0.13
	0.04	0.41		18	36	0.42
	0.15	0.53		15	30	0.51
	0.30	0.69		11	22	0.65
	2.48	0.81		6	12	0.81
BPEB$(OC_8H_{17})_2$	0.01	0.31	150	49	98	0.35
	0.02	0.42		40	80	0.47
	0.12	0.57		30	60	0.60
	0.38	0.71		22	44	0.71
	0.72	0.84		14	28	0.81
	17	0.92		6	12	0.92

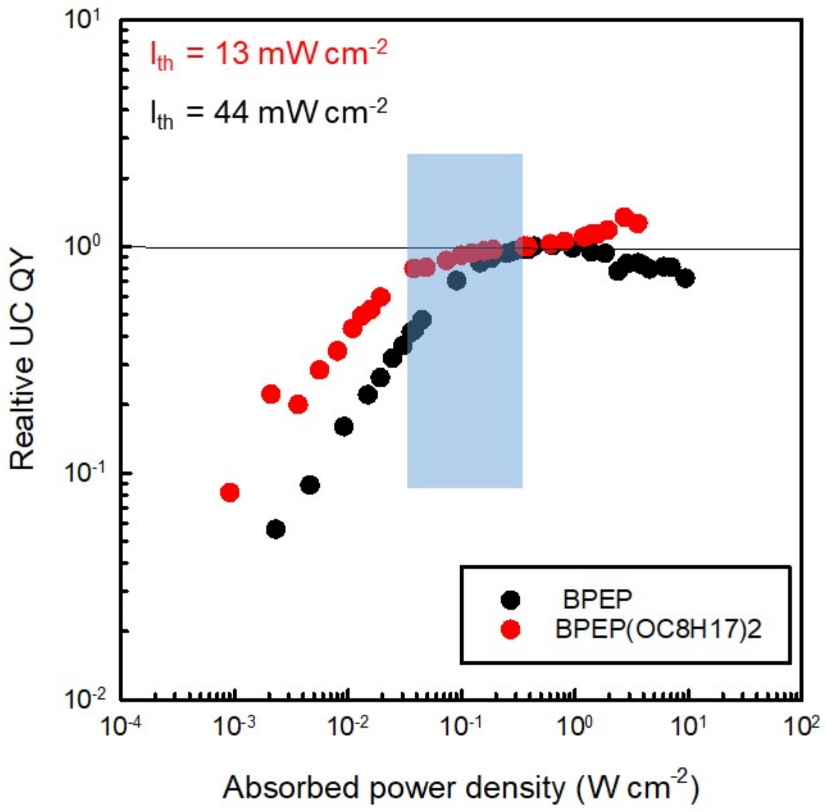

Figure S8. Normalized relative UC quantum yield for the investigated solutions. The TTA-UC intensity threshold can be estimated as the intensity at which 0.5 of the maximum yield is reached. This analysis affords threshold values that are in good agreement with those derived from the quadratic-to-linear fit of the UC emission intensity data shown in Fig. 4, within the uncertainty associated with these measurements. The shaded area marks the excitation regime considered to evaluate the average *f* factor for the TTA process.

4. Ultrafast transient absorption kinetic analysis

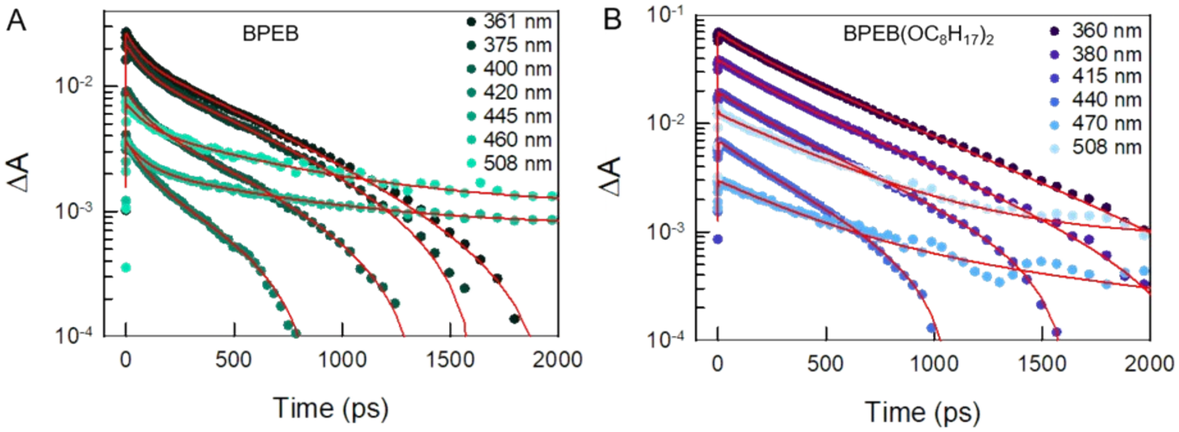

Figure S9. Ultrafast transient absorption (TA) kinetics recorded under a 340 nm pump, monitoring the absorbance change ΔA employing a UV probe, relative to BPEB (panel A) and to BPEB(OC_8H_{17})$_2$ (panel B), for chosen wavelengths reported in the legend. The negative kinetics were turned positive for clarity. The red lines are the fitting curves of the experimental data according to the sum of two or three decay components. The results of the fitting procedures are reported in Table S1 and S2. Some kinetic traces also account for a "long component", which refers to a decay component too long to be detected on the time scale afforded by these measurements (i.e., longer than few ns).

Table S2. Amplitudes a_i and rise and decay time constants resulting from the fitting of the ΔA kinetics under UV probe and 340 nm pump reported in Figure S9 (A) of BPEB.

**Wavelength [nm]**	a_1	IRF [ps]	a_2	$\tau_{decay,1}$ [ps]	a_3	$\tau_{decay,2}$ **[ps]**
361	0.06	0.17	0.01	61.48	0.02	541.7
375	0.04	0.21	0.009	76.5	0.01	630.12
400	0.02	0.13	0.004	86.58	0.006	652.22
420	0.01	0.15	0.002	86.62	0.003	699.42
460	0.005	0.26	0.001	85.17	0.002	737.8 + long component
508	0.008	0.44	0.003	72.8	0.004	573.38 + long component

Table S3. Amplitudes a_i and rise and decay time constants resulting from the fitting of the ΔA kinetics under UV probe and 340 nm pump reported in Figure S9 (B) of $BPEB(OC_8H_{17})_2$.

**Wavelength [nm]**	a_1	IRF [ps]	a_2	$\tau_{decay,1}$ **[ps]**	a_3	$\tau_{decay,2}$ **[ps]**	a_4	$\tau_{decay,3}$ **[ps]**
360	0.07	0.18	0.007	0.95	0.02	161.45	0.05	515.81
380	0.05	0.21	0.001	4.58	0.01	174.73	0.21	517.88
415	0.03	0.23	0.002	3.62	0.01	247.19	0.01	709.4
440	0.02	0.26	0.001	4.05	0.003	190.99	0.005	590
470	0.002	0.37			0.002	410.14	0.009	4376
508	0.01	0.29			0.001	6.87	0.01	453.78 + long component

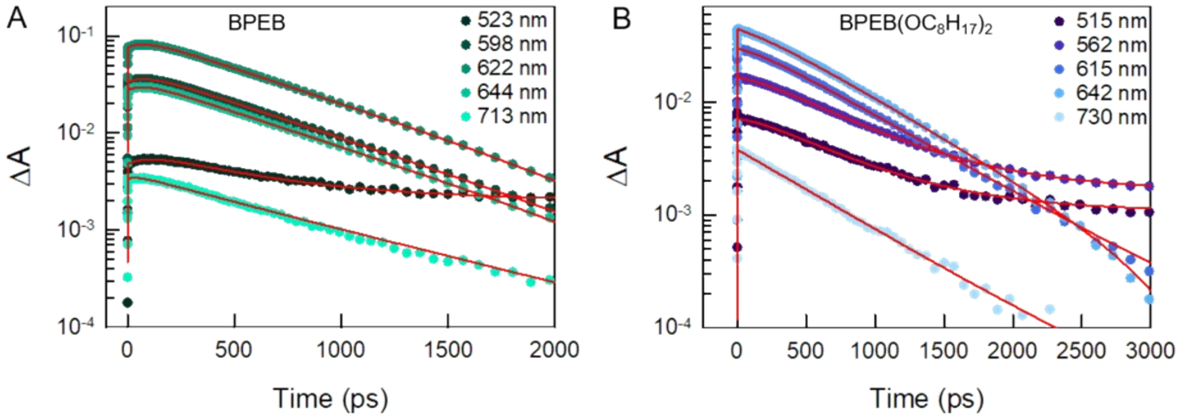

Figure S10. Ultrafast transient absorption (TA) kinetics recorded under a 340 nm pump, monitoring the absorbance change ΔA employing a visible probe, relative to BPEB (panel A) and to $BPEB(OC_8H_{17})_2$ (panel B), for chosen wavelengths reported in the legend. The red lines are the fitting curves of the experimental data according to the sum of a rising component and one or two decay components. The results of the fitting procedures are reported in Table S3 and S4. Some kinetic traces also account for a "long component", which refers to a decay component too long to be detected on the time scale afforded by these measurements (i.e., longer than few ns).

Table S4. Amplitudes a_i and rise and decay time constants resulting from the fitting of the ΔA kinetics under VIS probe and 340 nm pump reported in Figure S10 (A) of BPEB.

**Wavelength [nm]**	a_1	IRF [ps]	a_2	τ_{rise} [ps]	a_3	$\tau_{decay,1}$ [ps]	a_4	$\tau_{decay,2}$ **[ps]**
523	0.005	0.38	0.002	102.21	0.005	542.51		long component
598	0.04	0.43	0.01	88.78	0.05	599.89		long component
622	0.08	0.5	0.03	84.96	0.1	607.9		
644	0.03	0.49	0.01	75.48	0.04	610.36		
713	0.003	0.59	0.0005	43	0.002	611.05	0.002	1027

Table S5. Amplitudes a_i and rise and decay time constants resulting from the fitting of the ΔA kinetics under VIS probe and 340 nm pump reported in Figure S10 (B) of BPEB(OC_8H_{17})$_2$.

**Wavelength [nm]**	a_1	IRF [ps]	a_2	τ_{rise} [ps]	a_3	$\tau_{decay,1}$ [ps]	a_4	$\tau_{decay,2}$ **[ps]**
515	0.022	0.6	0.002	172.68	0.02	0.87	0.008	627.65 + long component
562	0.02	0.41	0.005	199.36	0.02	628.72		long component
615	0.03	0.54	0.007	176.15	0.04	637.28		
642	0.05	0.62	0.01	235.78	0.06	612.36		
730	0.004	0.42			0.004	611.73		

5. Transient absorption spectra analysis

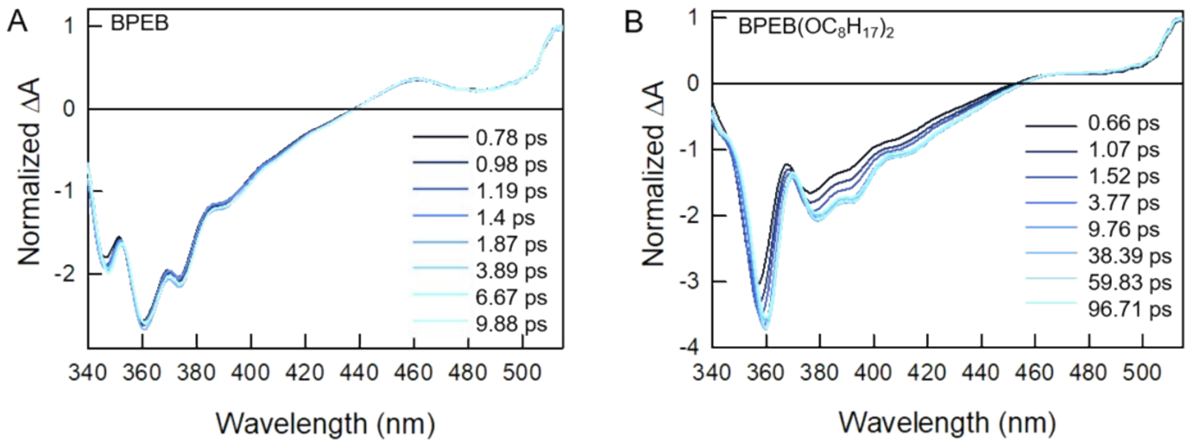

Figure S11. Transient absorption spectra at selected time delays recorded under a 340 nm pump, monitoring the absorbance change ΔA employing a UV probe (panel B), relative to BPEB (A) and to BPEB(OC_8H_{17})$_2$ (B), normalized to the maximum of the PIA feature, to highlight the redshift of the GSB/SE feature observed in the time intervals 0-10 ps (BPEB) and 0-100 ps (BPEB(OC_8H_{17})$_2$).

6. Synthesis

1-Iodo-4-(octyloxy)benzene. The synthesis was carried out as previously reported.[6] 4-Iodophenol (4.00 g, 13.6 mmol), 1-bromooctane (3.6 mL, 20.4 mmol), K_2CO_3 (5.6 g, 41 mmol) and DMF (45.4 mL) were added to a 100 mL round bottom flask and the mixture was stirred at room temperature for 10 min. The mixture was then heated to 80 °C and stirred overnight (o.n., ~ 14 h). The mixture was cooled down to room temperature and diluted with DCM (30 mL). The organic layer was washed with H_2O (4x20 mL), dried over Na_2SO_4 and collected. The solvent was removed under reduced pressure. Flash chromatography on silica gel was used to purify the crude product with n-hexane as eluent phase to afford the final product as yellow oil (4.2 g, 93% yield).

^{1}H-NMR (400 MHz, $CDCl_3$): δ 7.53 (d, J = 9.0 Hz, 2H), 7.26 (s, $CDCl_3$), 6.67 (d, J = 9.0 Hz, 2H), 3.91 (t, J = 6.6, 2H), 1.76 (m, 2H), 1.44 (m, 2H), 1.31 (m, 8H), 0.89 (t, J = 6.5 Hz, 3H).
^{13}C-NMR (100 MHz, $CDCl_3$): δ 159.03 (1C), 138.15 (2C), 116.95 (2C), 82.41 (1C), 68.15 (1C), 31.84 (1C), 29.36 (1C), 29.26 (1C), 29.18 (1C), 26.03 (1C), 22.68 (1C), 14.13 (1C).

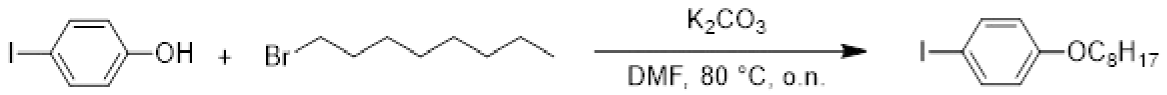

Figure S12. Reaction scheme for the synthesis of 1-iodo-4-(octyloxy)benzene.

1,4-Bis((4-(octyloxy)phenyl)ethynyl)benzene - BPEB(OC_8H_{17})$_2$

The synthesis was carried out as previously reported.[7] 1,4-Diethynylbenzene (300 mg, 2.38 mmol), 1-iodo-4-(octyloxy)benzene (5.24 mmol), Pd(PPh3)$_4$ (30 mg), and CuI (5 mg) were added to a 3:1 v/v mixture of toluene and diisopropylamine (40 mL) under inert atmosphere (N_2). The mixture was stirred and heated to 80 °C, and a precipitate started to form. The mixture was stirred for 18 h at 80 °C under N_2. The suspension was cooled to room temperature and then slowly dropped into an excess of MeOH (300 mL). The precipitate was filtered off over a glass filter with porosity P4 and washed with toluene (3x20 mL). The solvents were then removed under reduced pressure to afford an orange/brown solid. The crude product was purified with an automatic liquid column chromatography Biotage Isolera^{TM} One equipped with the BGB Scorpius Flash Cartridge (BSS2CF-W080), silica 60 Å, using a gradient eluent phase of n-Hexane-$CHCl_3$. The solvents were then removed under reduced pressure to afford yellowish plate-shaped crystals. The crystals were finally recrystallized from DCM to afford pure white plate-shaped crystals (0.24 g, 20%).

^{1}H-NMR (400 MHz, $CDCl_3$): δ 7.52 (d, J = 8.8 Hz, 4H), 7.42 (s, 4H), 7.16 (s, benzene-D_6), 6.72 (d, J = 8.8 Hz, 4H), 3.55 (t, J = 6.5 Hz, 4H), 1.58 (dq, J = 8.3, 6.5 Hz, 4H) 1.28 (m, 20H), 0.92 (t, J = 7.0, 6H).

^{13}C-NMR (100 MHz $CDCl_3$): δ 159.37 (2C), 133.05 (4C), 131.30 (4C), 123.09 (2C), 114.91 (3C), 114.58 (3C), 91.27 (2C), 87.87 (2C), 68.12 (2C), 31.81 (2C), 29.35 (2C), 29.23 (2C), 29.20 (2C), 26.03 (2C), 22.66 (2C), 14.10 (2C).

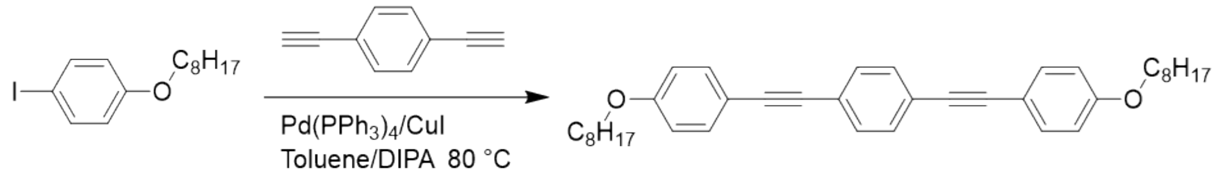

Figure S13. Reaction scheme for the synthesis of BPEB(OC_8H_{17})$_2$.

7. NMR spectra

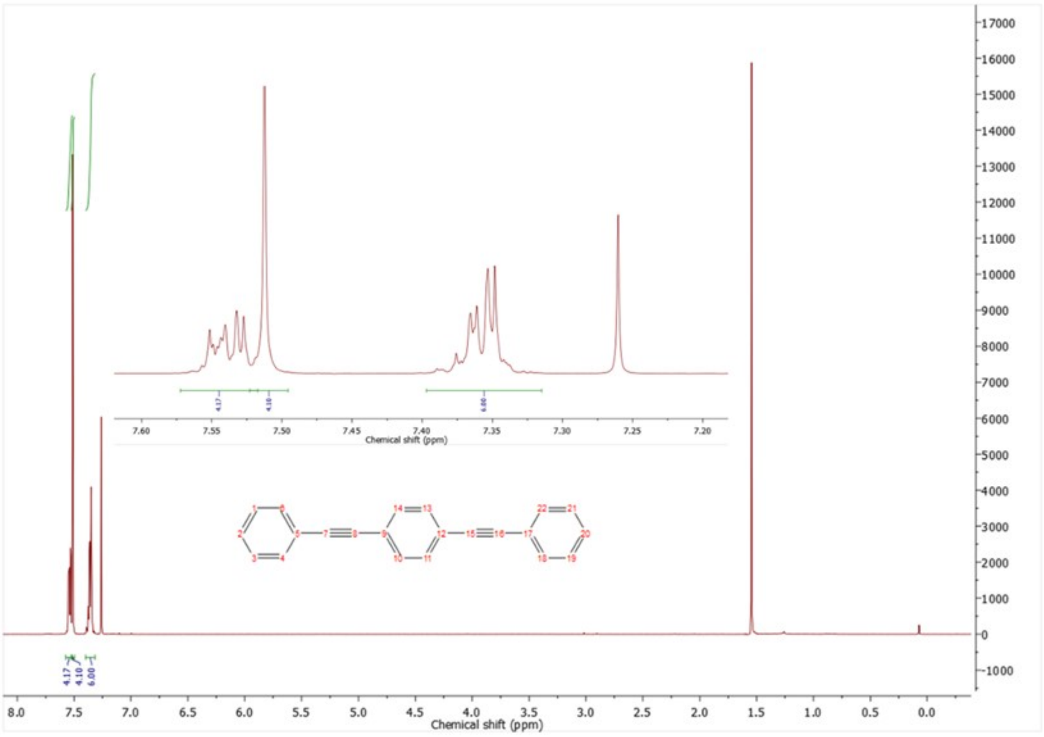

Figure S14. ^{1}H-NMR spectrum (400 MHz) of BPEB in $CDCl_3$.

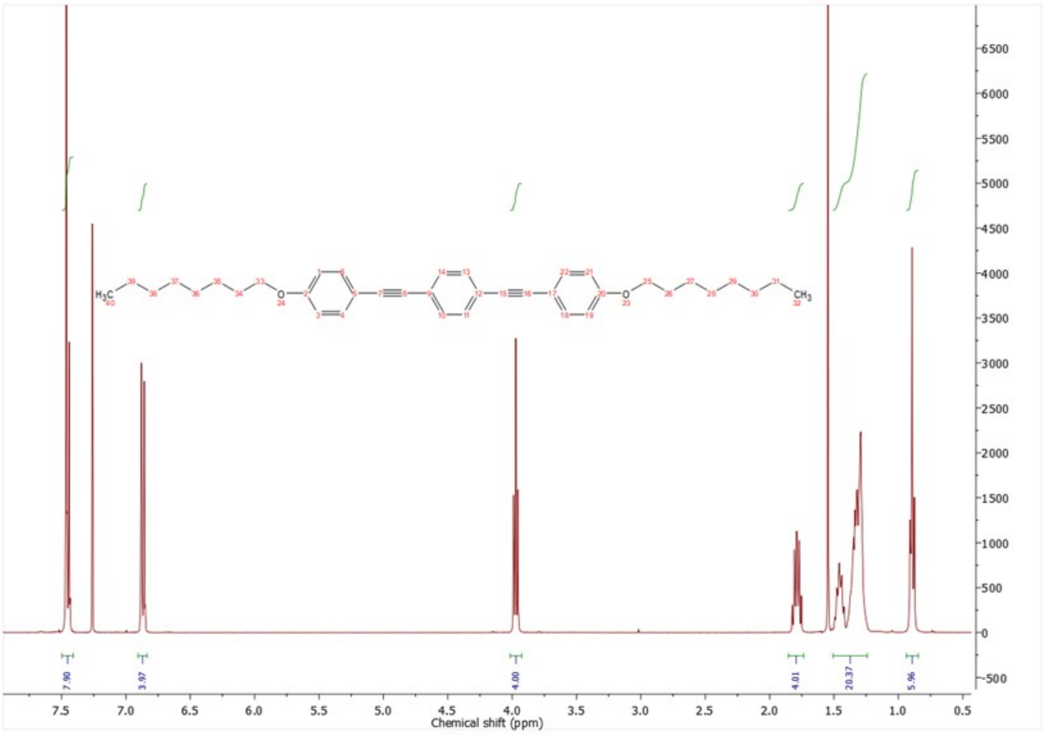

Figure S15. ^{1}H-NMR spectrum (400 MHz) of $BPEB(OC_8H_{17})_2$ in $CDCl_3$.

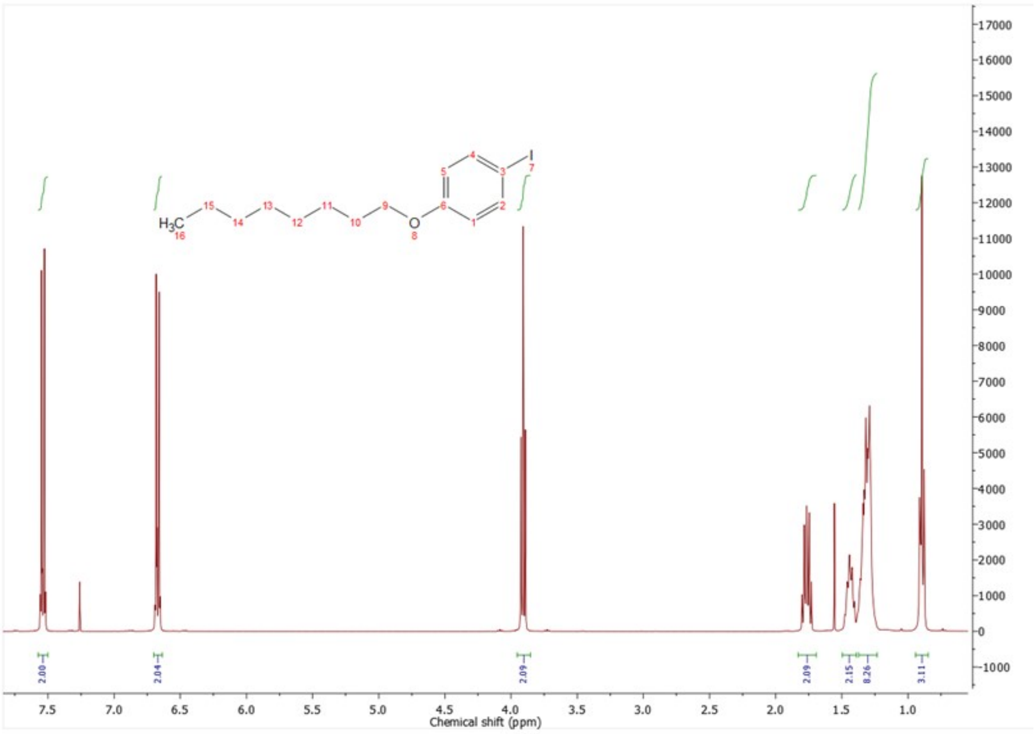

Figure S16. ¹H-NMR spectrum (400 MHz) of 1-iodo-4-(octyloxy)benzene in $CDCl_3$.

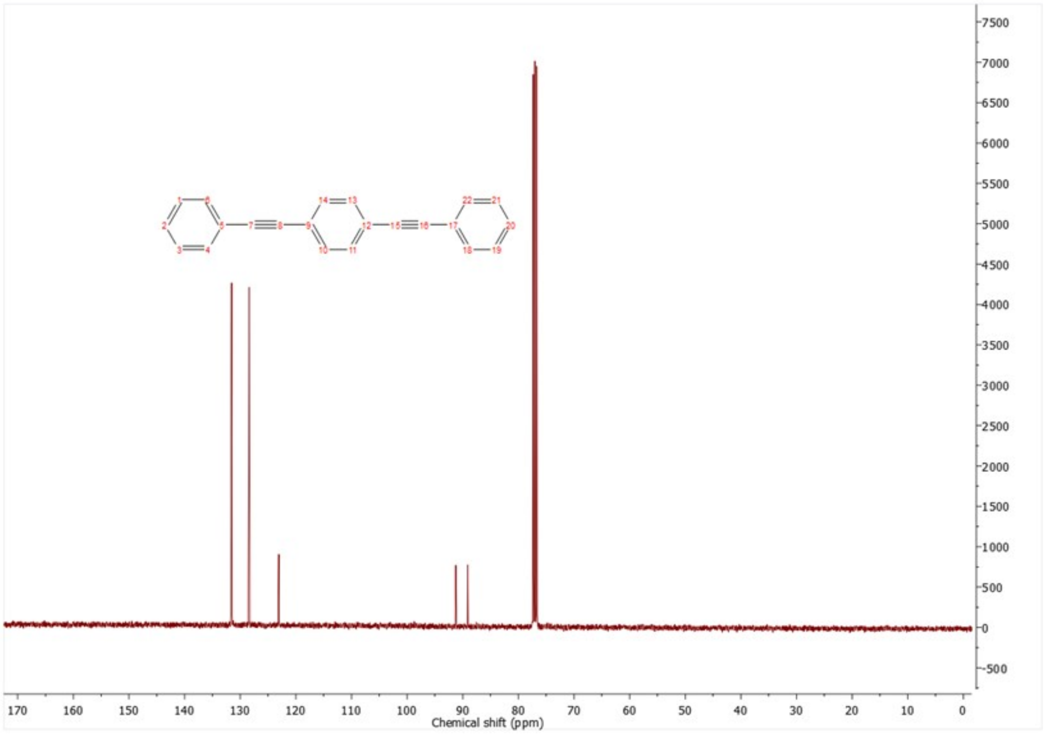

Figure S17. ¹³C-NMR spectrum (100 MHz) of BPEB in $CDCl_3$.

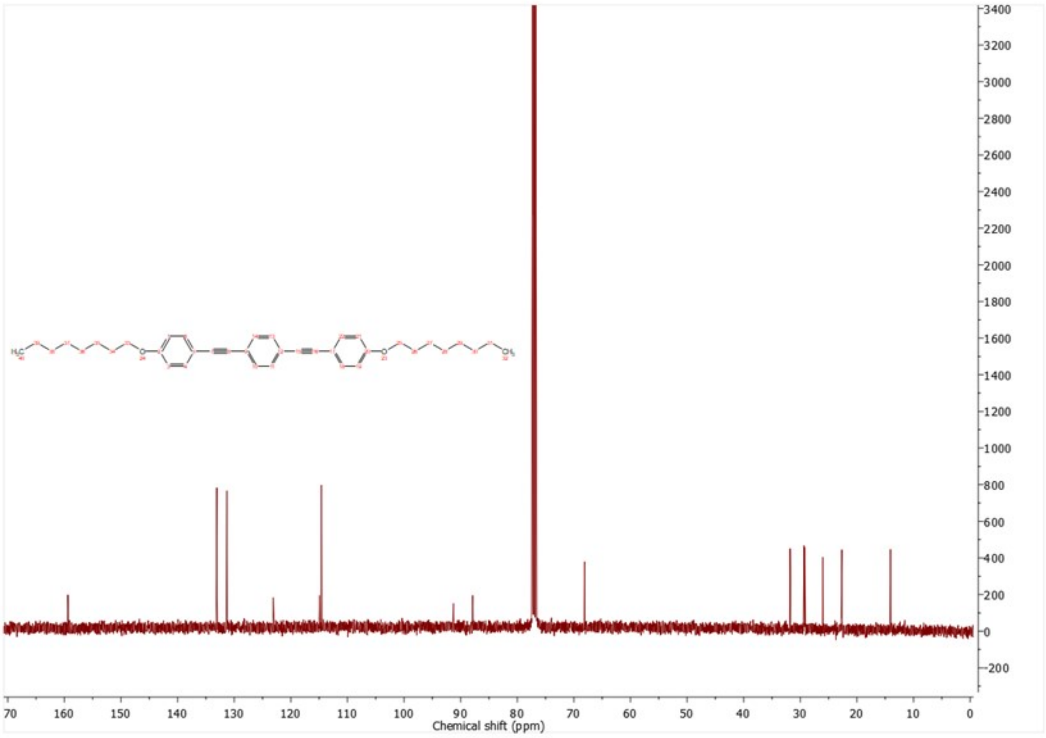

Figure S18. ^{13}C-NMR spectrum (100 MHz) of BPEB(OC_8H_{17})$_2$ in $CDCl_3$.

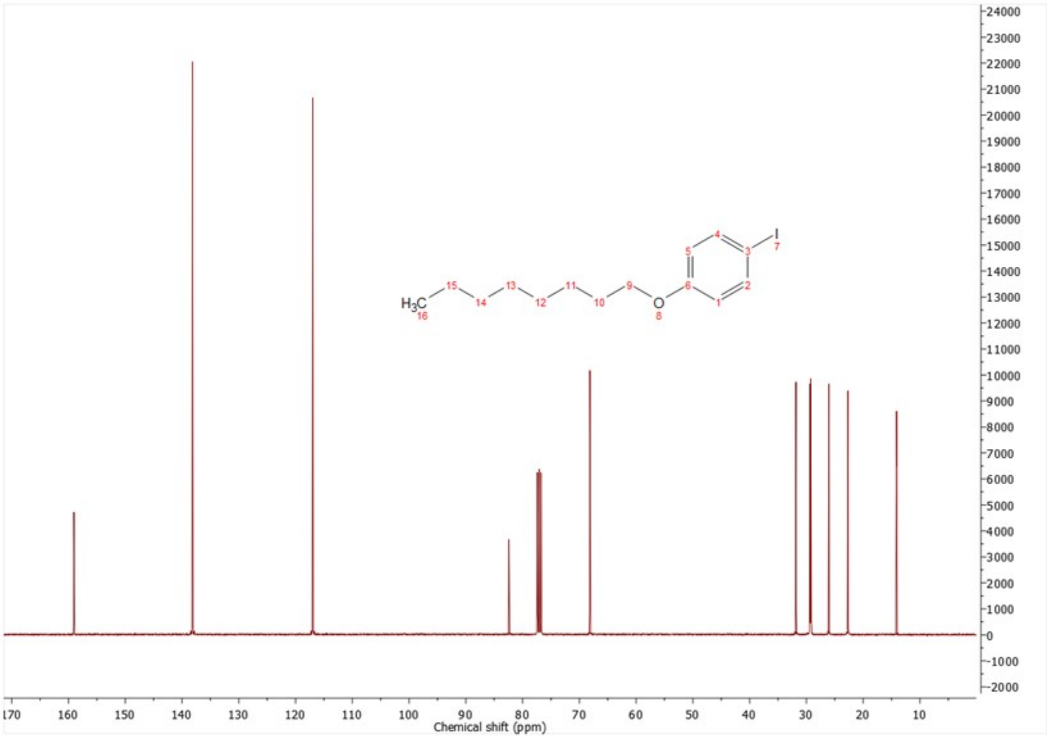

Figure S19. ^{13}C-NMR spectrum (100 MHz) of 1-Iodo-4-(octyloxy)benzene in $CDCl_3$.

8. Reference Vis-to-UV photon upconversion system

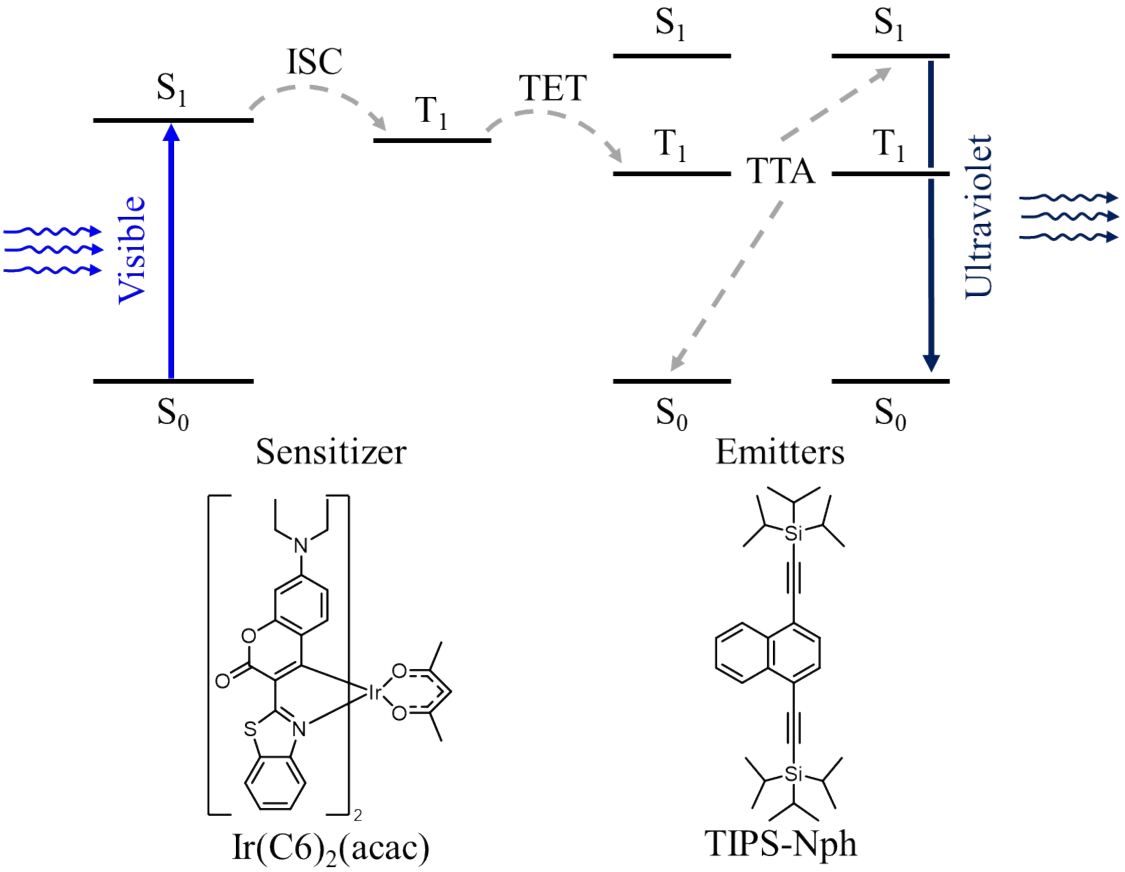

Figure S20. Schematic representation of the visible-to-ultraviolet triplet–triplet annihilation upconversion (TTA-UC) process with Ir(C6)(acac) (sensitizer) and TIPS-NPh (annihilator). Upon visible light excitation, the sensitizer transitions from its ground state (S_0) to the singlet excited state (S_1), followed by intersystem crossing (ISC) to the triplet excited state (T_1). The triplet energy is transferred to an annihilator through triplet energy transfer (TET). Two annihilator triplets then undergo TTA, resulting in the population of a high-energy singlet excited state (S_1) of the annihilator, which emits ultraviolet photons upon relaxation to the ground state. Solid arrows indicate radiative processes while dashed arrows indicate non-radiative processes.

Measurements were performed with a Hamamatsu C10083CA spectrometer using a 473 nm laser as a pump source.

Excitation wavelength	473 nm
Integration time	5 ms
Excitation density	22.04 W/cm^2 (5.25e19 ph/cm^2)

The Tips-Nph + Ir complex sample in a sealed solution in THF lost 30% of its upconverted fluorescence quantum yield after 2000 s of irradiation (Fig. S21). A UV transmitting black glass was used as high-pass filter.

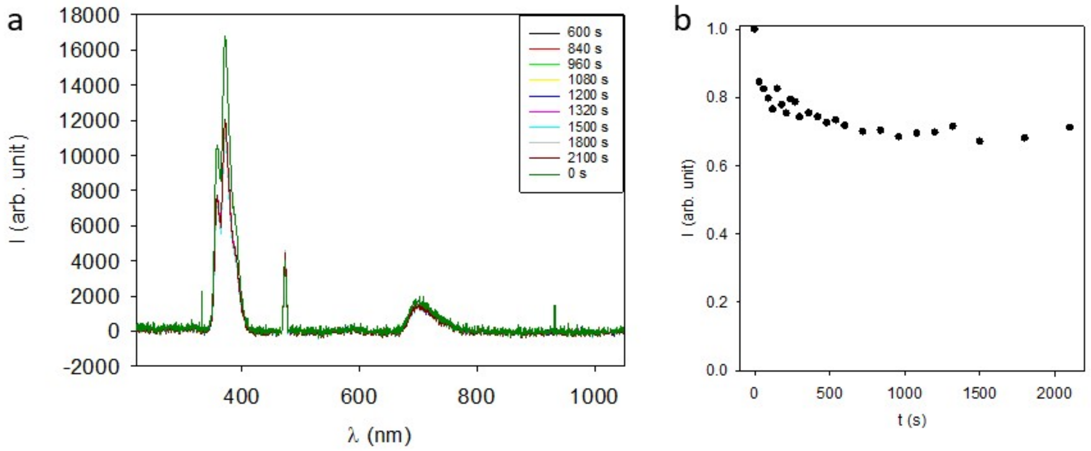

Figure S21. Upconverted fluorescence spectra (a) of a Ir complex + Tips-Nph sample in a THF solution (10^{-5} M, 10^{-3} M) and (b) integrated UC emission intensity at 380 nm measured as a function of time under 473 nm laser excitation.

9. Stability Test

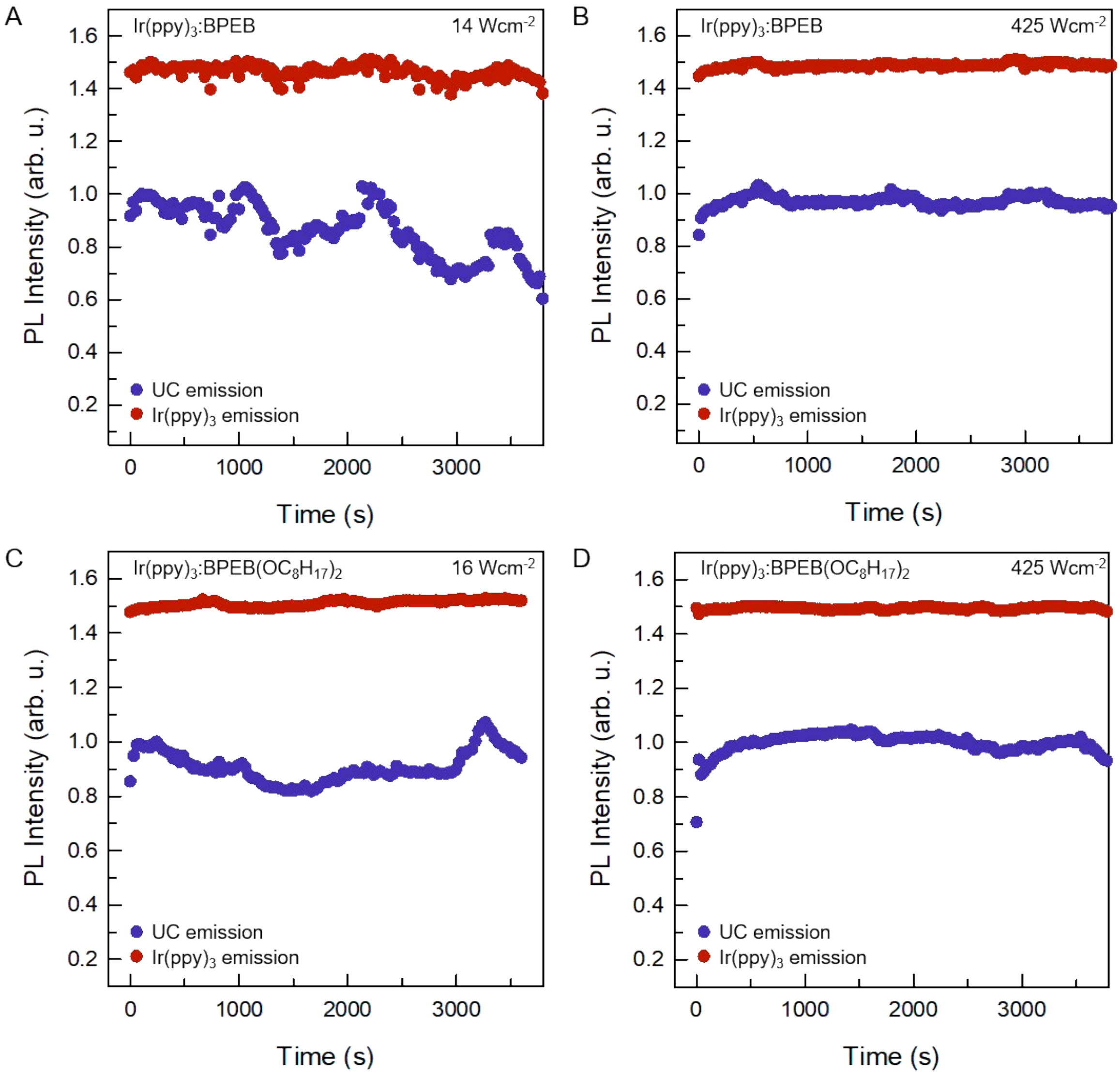

Figure S22. Time evolution of the integrated upconversion (blue dots) and residual sensitizer (red dots) emission intensity, for $Ir(ppy)_3$:BPEB (panels A, B) and the $Ir(ppy)_3$:BPEB(OC_8H_{17})$_2$ (panels C and D) solutions (sensitizer concentration 2×10^{-4} M, emitters concentration 5×10^{-3} M), recorded under continuous excitation up to 4500 s, using a 473 nm cw laser as excitation source. Panels A and C report measurements at a power density slightly above the threshold intensity, while in B and D the power density is much above the threshold intensity (Panel A – 3.31e19 ph/cm^2, panel B – 1.01e21 ph/cm^2, panel C – 3.71e19 ph/cm^2, panel D – 1.01e21 ph/cm^2).

10. Supporting references

(1) Kawaoka, K.; Khan, A. U.; Kearns, D. R. Role of Singlet Excited States of Molecular Oxygen in the Quenching of Organic Triplet States. *The Journal of Chemical Physics* **1967**, *46* (5), 1842-1853.

(2) Kearns, D. R.; Stone, A. J. Excited-State Intermolecular Interactions Involving Paramagnetic Molecules: Effect of Spin—Spin and Spin—Orbit Interactions on the Quenching of Triplets. *The Journal of Chemical Physics* **1971**, *55* (7), 3383-3389.

(3) Berlman, I. B. *Handbook of Fluorescence Spectra of Aromatic Molecules*; Academic Press, 1971.

(4) Suzuki, K.; Kobayashi, A.; Kaneko, S.; Takehira, K.; Yoshihara, T.; Ishida, H.; Shiina, Y.; Oishi, S.; Tobita, S. Reevaluation of absolute luminescence quantum yields of standard solutions using a spectrometer with an integrating sphere and a back-thinned CCD detector. *Physical Chemistry Chemical Physics* **2009**, *11* (42), 9850-9860, 10.1039/B912178A.

(5) Sajoto, T.; Djurovich, P. I.; Tamayo, A. B.; Oxgaard, J.; Goddard, W. A., III; Thompson, M. E. Temperature Dependence of Blue Phosphorescent Cyclometalated Ir(III) Complexes. *Journal of the American Chemical Society* **2009**, *131* (28), 9813-9822.

(6) Rondeau-Gagné, S.; Curutchet, C.; Grenier, F.; Scholes, G. D.; Morin, J.-F. Synthesis, characterization and DFT calculations of new ethynyl-bridged C60 derivatives. *Tetrahedron* **2010**, *66* (23), 4230-4242.

(7) Palmans, A. R. A.; Eglin, M.; Montali, A.; Weder, C.; Smith, P. Tensile Orientation Behavior of Alkoxy-Substituted Bis(phenylethynyl)benzene Derivatives in Polyolefin Blend Films. *Chemistry of Materials* **2000**, *12* (2), 472-480.
